# Robust Classification of Small-Molecule Mechanism of Action Using a Minimalist High-Content Microscopy Screen and Multidimensional Phenotypic Trajectory Analysis

**DOI:** 10.1371/journal.pone.0149439

**Published:** 2016-02-17

**Authors:** Nathaniel R. Twarog, Jonathan A. Low, Duane G. Currier, Greg Miller, Taosheng Chen, Anang A. Shelat

**Affiliations:** Department of Chemical Biology and Therapeutics, St. Jude Children’s Research Hospital, Memphis, Tennessee, United States of America; Stanford University, UNITED STATES

## Abstract

Phenotypic screening through high-content automated microscopy is a powerful tool for evaluating the mechanism of action of candidate therapeutics. Despite more than a decade of development, however, high content assays have yielded mixed results, identifying robust phenotypes in only a small subset of compound classes. This has led to a combinatorial explosion of assay techniques, analyzing cellular phenotypes across dozens of assays with hundreds of measurements. Here, using a minimalist three-stain assay and only 23 basic cellular measurements, we developed an analytical approach that leverages informative dimensions extracted by linear discriminant analysis to evaluate similarity between the phenotypic trajectories of different compounds in response to a range of doses. This method enabled us to visualize biologically-interpretable phenotypic tracks populated by compounds of similar mechanism of action, cluster compounds according to phenotypic similarity, and classify novel compounds by comparing them to phenotypically active exemplars. Hierarchical clustering applied to 154 compounds from over a dozen different mechanistic classes demonstrated tight agreement with published compound mechanism classification. Using 11 phenotypically active mechanism classes, classification was performed on all 154 compounds: 78% were correctly identified as belonging to one of the 11 exemplar classes or to a different unspecified class, with accuracy increasing to 89% when less phenotypically active compounds were excluded. Importantly, several apparent clustering and classification failures, including rigosertib and 5-fluoro-2’-deoxycytidine, instead revealed more complex mechanisms or off-target effects verified by more recent publications. These results show that a simple, easily replicated, minimalist high-content assay can reveal subtle variations in the cellular phenotype induced by compounds and can correctly predict mechanism of action, as long as the appropriate analytical tools are used.

## Introduction

Understanding of a compound’s mechanism of action is essential to the development and evaluation of small molecule therapeutics. This is obviously true for novel therapeutic candidates and elements of large natural product or bioactive screening libraries, but just as critical for more developed and established therapeutics, as even clinically available compounds can produce unexpected secondary or off-target effects.[1] Phenotypic assays are increasingly being used to evaluate compounds in a more complete cellular or tissue microenvironment. [2] High content screening (HCS) in particular carries great potential for drug discovery and development, combining the historic breadth and versatility of visual microscopy with the power, speed, and efficiency of automated screening.[3,4] Yet the apparent potential of HCS has remained frustratingly unrealized: in their 2014 review of multidimensional small molecule profiling, Wolpaw and Stockwell devoted only one page of thirty-five to quantitative imaging, concluding that “only a minority of compounds displayed an appreciable phenotype”.[5] Despite the lack of progress in the field, we feel that many potential insights remain untapped in the analysis and representation of HCS data, and such findings can be unlocked with improved analytical methods.

The challenges of HCS analysis are numerous. A typical HCS experiment might produce thousands of images, terabytes of data, and gigabytes of extracted cellular measurements. Some of these measurements, such as average α-tubulin intensity and cellular size, have clear biological significance; whether the additional dozens or hundreds of dimensions of measurements extracted by a HCS image-processing pipeline contain further phenotypic insights, however, is a difficult question to answer. Even if statistically significant variations in measured phenotype can be identified, the dimensions involved are often inscrutable mixed bags of correlated measurements that offer little concrete insight into the effects of a given compound or mechanism of action in a cell; this is increasingly true as the number and granularity of measurements and stains increases. Furthermore, several studies have shown that important variations in cellular phenotype are produced by different doses or concentrations of many compounds,[6,7] but measurement of similarity between dose response patterns—ordered sequences of cellular phenotypic distributions—is a complex, subjective problem, which does not lend itself to many traditional out-of-the-box measures of similarity.[8]

Though several groups have had considerable success adapting HCS to the identification or detection of certain pre-selected phenotypes,[9–11] such approaches are not always sufficient; after all, a secondary screen for off-target effects is hardly necessary if one knows what effects to look for. Broader, more phenotypically agnostic methods have met with only limited success, however, often identifying only a small number of phenotypic classes such as microtubule inhibitors or stabilizers.[6,12] Yet the ubiquitous influence and application of visual microscopy clearly attest that deeper biological insights lay buried in the ever increasing haystack of high content data. We therefore set ourselves the following challenge: given a simple, standardized, minimalist high content assay, using three basic stains and roughly two dozen cellular measurements, how well can we understand and classify the mechanism of action of a diverse set of active compounds? In short, how much information about mechanism of action can be extracted from a minimalist HCS assay? To address this question, we investigated the impact of three analytical choices: the method of dimensional reduction for visualization and analysis, the use of dose-response data over single-point measurements, and the appropriate measure of inter-compound similarity.

This paper describes our analytical approach, derived in answer to these three concerns. First, though most approaches to high-content data use principal components analysis (PCA) or common factor analysis [13,14] to perform the dimensionality reductions necessary for visualization—and in some cases, analysis—of high content data, we have found that use of multi-class linear discriminant analysis (LDA) [15] produces dimensions that are far more informative, and produce more intuitive visualizations with improved classification accuracy. Second, though single-point screens may sometimes be necessitated by pragmatic considerations, our analyses show that use of dose response data not only improves accuracy in clustering and classification, but allows for deeper, more intuitive insights that are fundamentally inaccessible from single-point data. Several mechanistic classes, including topoisomerase inhibitors and histone deacetylase (HDAC) inhibitors, produced highly distinct phenotypic behaviors only visible across a range of doses. Finally, we found that an effective measure of inter-compound similarity, particularly with dose-response data, should be titration- and dilution-independent, and should ignore phenotypic similarities between populations that are themselves similar to untreated cells. Using this analytical paradigm, we were able to identify a number of phenotypic clusters, several of which have not been noted in previous phenotypic screens, and develop an approach to the phenotypic classification of compound behavior that reliably recognizes almost a dozen distinct mechanistic classes.

## Materials and Methods

### Preparation of Cells, Staining, Capturing Images, and Processing Images

300 HeLa cells in 25 μl of media were plated into each well of a poly-D-lysine coated Perkin Elmer 384-well View plates (Perkin Elmer 6007710) with a Thermo Scientific Wellmate, in triplicate plates. The cells were cultured for 18 hours before the addition of compounds by a VP scientific pintool fitted with FP3S100 pins. Compounds were incubated with cells for forty-eight hours. Following treatment, the cells were fixed with 4% formaldehyde for 20 minutes at 37°C, then washed with 75 μl of PBS 3 times. The cells were permeabilized with 0.1% Triton X-100 for 15 minutes at 25°C and blocked using 1% BSA in PBS for 1 hour at 25°C. Primary antibodies against p-H2A.X (Ser139) (Cell Signaling 9718) and α-tubulin (Sigma T6199) were diluted 1:200 and 1:1000, respectively, in 1% BSA in PBS and incubated with the fixed cells overnight at 4°C. The plates were washed 3 times with PBS using a Biotek 405LS plate washer, and incubated for 1 hour at 25°C with a solution containing goat α-rabbit-Alexa-488 (Cell Signaling 4412S) and goat α-mouse-Alexa-647 (Cell Signaling 4410S) secondary antibodies each diluted 1:400 in 1% BSA in PBS in addition to 1 μM Hoechst 34580 (Molecular Probes H21486). Images were captured using a GE Healthcare InCell 6000 equipped with a 4X objective lens with the capability of imaging the entire well in a single acquisition. Fluorescent emissions at 405 nm, 488 nm, and 647 nm were captured to detect nuclear staining, p-H2A.X, and α-tubulin, respectively. All data were analyzed using the multi-target analysis algorithm of the GE InCell Analyzer Workstation software. The number of nuclear objects in each well and all nuclear measurements were calculated by masking the nucleus based on Hoechst staining. Phospho-H2A.X intensity was measured within each nuclear mask. Cell size and shape parameters were measured by masking the cytoplasm based on the α-tubulin channel.

### Preparation of Compounds

We identified 160 compounds from our local chemical inventory that represent diverse mechanisms of action relevant to mammalian cellular biology, including anti-metabolites, apoptosis inducers, DNA damaging agents, epigenetic modifiers, kinase inhibitors, microtubule inhibitors, mitochondrial poisons, and proteasome inhibitors. Where possible, we chose at least three representatives from each of the broadly defined classes listed above. We also included a few idiosyncratic compounds such as auranofin, bardoxolone methyl, and ouabain that often show up as active compounds in cell-based screens and demonstrate complex mechanistic behavior. Six compounds were unavailable in sufficient amount and were excluded from this study, resulting in a training set of 154 compounds. Compounds were obtained as dry powder and dissolved in DMSO to a target concentration of either 10mM or 2mM (for compounds with reported high cellular potency or solubility limitations). The purity of each compound was verified to be >95% using UV/TWC and evaporative light scattering detection (ELSD) spectroscopy, and the concentration of each solution was quantified using nitrogen chemo-luminescence where possible. Each compound was then serially diluted into a 384-well polypropylene dose-response plate with three-fold dilution to give 10 concentration points per compound (32 compounds per plate). Approximately 140nl of compound stock solution was transferred to the assay plate using a pintool as described earlier (178-fold dilution from stock concentration).

### Dimensional Analysis

Though all compounds were tested at 10 concentrations separated by a 3-fold serial dilution, the highest concentrations often produced far noisier, more divergent phenotypes, perhaps due to edge effects, liquid handling artifacts, or compound precipitation. The response to the highest concentration of each compound was excluded from analysis. Additionally, 12 wells in the experiment exhibited mean Hoechst stain intensity well above the observed distribution; visual inspection revealed that these wells were contaminated, either by UV fluorescing artifacts or by the presence of unexplained biological growth in the well. These wells were also eliminated from the analysis.

From the 47 measurements extracted in the image analysis, several that were deemed not relevant to the current analysis (e.g. a cell’s location within the well) or that were direct calculations from other measurements (e.g. total intensity, which is simply the product of area and average intensity) were discarded. Measurements of pH2A.X intensity were normalized to background levels of 488nm fluorescence in all wells (see Results). The remaining 23 measurements were transformed to yield more normal distributions; most, including measurements of size, area, and intensity were log transformed, while several shape factor measurements which must lie between 0 and 1 were passed through a logit transform. A full description of the 23 measurements used and how they were transformed can be found in S1 Table. Finally, to minimize inter-plate variation, all measurements were normalized to the mean value for control wells on the same plate by subtracting that mean value. For PCA, all dimensions were normalized to have a variance of 1.

Formally, multiclass LDA requires identifying the eigenvectors of $\Sigma_{w}{}^{-1}\Sigma_{b}$, where Σ_*w*_ is the average within-class covariance, and Σ_*b*_ is the average between-class covariance. Rather than use the variable, subjective, and potentially biasing breakdown of mechanism class available in the existing literature, we instead used as “classes” the different concentration levels of the 154 compounds included in the training plates (note that covariance across concentration levels is not part of within-class covariance, but part of between-class covariance). Thus, the extracted dimensions are those which best distinguish different compounds (or differing concentrations of the same compound) from one another. In traditional multiclass LDA, the number of classes is smaller than the number of measurement dimensions and the purpose of the analysis is to calculate the most informative subspace capturing all between class covariance; in our case, because the number of “classes” (that is, concentration levels of compounds) is much larger than the number of measurement dimensions, we are simply generating a ranking of subspaces, in which the *m* eigenvectors with the largest eigenvalues span the most informative *m*-dimensional subspace. Therefore, our particular variation of multi-class LDA can be considered *over-classed*. For comparison, an LDA analysis was also run in which all cells exposed to any treatment (any dose of any drug) in one of the 11 exemplar classes were aggregated into a single LDA class. Because these exemplar classes (as well as the null class of all unclassified compounds) only produce 12 classes for analysis, a maximum of 11 informative dimensions could be extracted.

### Phenotypic Similarity

Because the phenotypic responses in this assay consist of changing cellular distributions at different concentration levels, which we call *phenotypic trajectories*, any similarity measure must capture the overlap of such trajectories in phenotypic space. We thus take the TISS approach of Perlman *et al*. [6] one step further with the *maximum sequential weighted overlap* (*MSWO*), defined as follows: suppose *D*_1_ and *D*_2_ are two distributions with means *μ*_1_ and *μ*_2_ and covariance matrices Σ_1_ and Σ_2_; then the *overlap* between these two distributions is defined as

$$O\left(D_{1},D_{2}\right)=\frac{\sqrt{\left|4\Sigma_{1}\Sigma_{2}\right|}}{\left|\Sigma_{1}+\Sigma_{2}\right|}\text{exp}-\frac{1}{2}{\left(\mu_{1}-\mu_{2}\right)}^{t}{\left(\Sigma_{1}+\Sigma_{2}\right)}^{-1}\left(\mu_{1}-\mu_{2}\right)$$

This measure is equal to 1 if the two distributions are identical, but drops if the distributions are separated or have highly distinct covariance matrices. For two cellular distributions *D*_1_ and *D*_2,_ the *weighted overlap* between these two distributions is equal to

$$O_{W}\left(D_{1},D_{2}\right)=O\left(D_{1},D_{2}\right)\left(1-O\left(D_{1},D_{0}\right)\right)\left(1-O\left(D_{2},D_{0}\right)\right)$$

where *D*_0_ is the cellular distribution observed in control wells (which, because of plate normalization, will always have a mean of 0 for all measurements). This weighting ensures that similarity between phenotypes contributes to inter-compound similarity only if those phenotypes are highly different from that of untreated cells. Finally, if the two phenotypic trajectories for compounds *C*_*i*_ and *C*_*j*_ are represented by the sequences of cellular distributions (*D*_i,1_,…*D*_i,L_) and (*D*_j,1_,…*D*_j,L_), then the MSWO between the two trajectories is:

$$S_{W}\left(C_{I},C_{j}\right)=\frac{\underset{1\leq k\leq L}{\text{max}}\underset{1\leq m\leq L}{\text{max}}SO_{k,m}\left(C_{i},C_{j}\right)}{\sum_{k=1}^{L}N_{i,k}+\sum_{m=1}^{L}N_{j,m}}$$

where *N*_*i*,*k*_ and *N*_*j*,*m*_ are the number of cells left after exposure to compound *C*_*i*_ at concentration level *k* and compound *C*_*j*_ at concentration level *m* respectively, and

$$SO_{k,m}\left(C_{i},C_{j}\right)=\{\begin{array}{cc} k>1,m>1 & \left(N_{i,k}+N_{j,m}\right)O_{W}\left(D_{i,k},D_{j,m}\right)+\underset{k^{\prime }<k}{\text{max}}\underset{m^{\prime }<m}{\text{max}}SO_{k\prime ,m\prime }\left(C_{i},C_{j}\right)\\ k=1\;\text{or}\;m=1 & \left(N_{i,k}+N_{j,m}\right)O_{W}\left(D_{i,k},D_{j,m}\right) \end{array}$$

**Fig 1:**
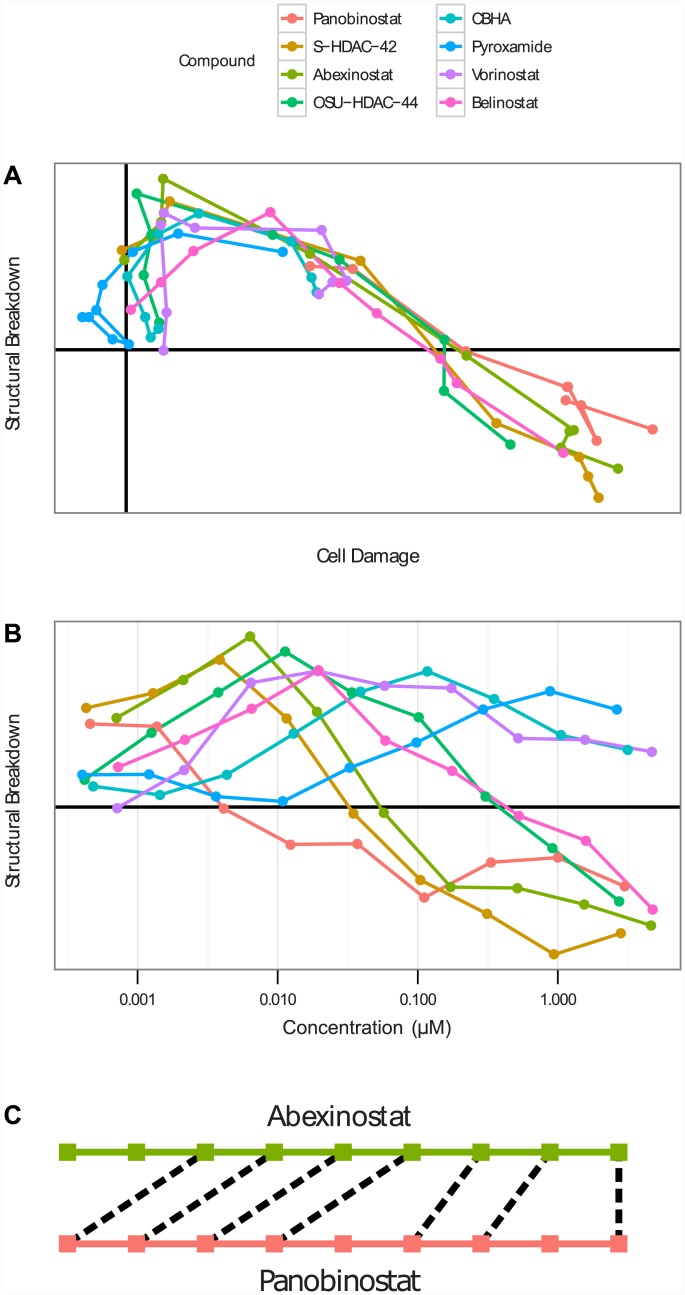
Maximum Sequential Weighted Overlap. **(A)** HDAC inhibitors follow a consistent, stereotypical phenotypic track, shown here in the “Cell Damage” and “Structural Breakdown” (tubulin intensity outside cell boundary, Hoescht stain outside nucleus, increased tetraploidy signal) dimensions. **(B)** Different compounds move through “Structural Breakdown” with differing potencies and sensitivities. **(C)** The optimal overlap between abexinostat and panobinostat uses the highest seven concentrations of abexinostat and a non-consecutive subset of doses of panobinostat.

In short, given two phenotypic trajectories represented by sequences of cellular phenotypic distributions, subsequences of equal length, not necessarily consecutive, of each trajectory are chosen such that the sum of the pairwise overlaps between corresponding cellular distributions in the two subsequences is maximized (Fig 1). The overlap between two cellular distributions is determined by the agreement of their means and covariances, and is weighted by how dissimilar those two distributions are from the distribution of untreated cells. This attenuation of distributions similar to untreated cells proves to be a critical element of an effective similarity measure. The MSWO can be calculated on cellular distributions of any dimensionality; unless otherwise specified, the results reported in this paper used the 16 most informative dimensions extracted by over-classed LDA (see Results).

Weighting a cellular distribution according to its dissimilarity from the distribution of untreated cells provides us with another useful measure: by taking the average dissimilarity from the distribution of untreated cells across a compound’s entire phenotypic trajectory, we have a measure of how phenotypically distinctive that compound is. We call this measure *phenotypic activity*, defined as follows: suppose a phenotypic trajectory for compound *C* is represented by a sequence of distributions (*D*_1_,…*D*_*L*_). Assuming that overlap *O* is defined as above, the *phenotypic activity* of *C* is defined as

$$A_{P}\left(C\right)=\frac{\sum_{k=1}^{L}N_{k}\left(1-O\left(D_{k},D_{0}\right)\right)}{\sum_{k=1}^{L}N_{k}}$$

where *D*_0_ is the cellular distribution of untreated cells, and *N*_*k*_ is the number of cells left after exposure to compound *C* at concentration level *k*.

### Hierarchical Clustering and Compound Classification

Of the 154 training compounds, 137 could be identified with one of 18 mechanistic classes; the remaining 17 compounds were too idiosyncratic to associate with a mechanism shared by other test compounds (see S2 and S3 Tables). To ensure the robustness of classification, only those mechanistic classes containing at least 4 compounds with a phenotypic activity above 0.4 were used as exemplar classes. Compounds in those classes exceeding the phenotypic activity threshold were used as exemplar compounds. This ensured that in cross-validation of the classification method, all compounds were compared with at least 3 phenotypically active exemplar compounds in each exemplar class. Several classes, such as mTOR inhibitors and tyrosine kinase inhibitors, were too small to include as exemplar classes, while others, including the alkylating agents, were far too phenotypically inactive to produce a robust classification.

Given the MSWO similarity scores for all pairs of compounds, hierarchical clustering of compounds was performed using UPGMA pairwise clustering.[16] For compound classification, the similarity between a test compound and a given mechanism class was simply the average of all similarity scores for all exemplar compounds in that class.

Because there is the considerable possibility that a given test compound will not belong to any exemplar compound class, a classification method should account for this possibility. All 154 compounds were compared against the 85 exemplar compounds to determine their maximum mechanism class similarity (“Best Match”); the distributions of these maximum similarities for classified and unclassified compounds, normalized to each compound’s phenotypic activity, were compared, and Bayes’ rule was used to determine a threshold below which the maximum a posteriori (MAP) likelihood was greater that a compound did not belong to one of the 11 exemplar mechanism classes, as follows. Passing all normalized maximum similarities through a log transform generates a more normal distribution. The mean and variance of the distribution of transformed maximum similarities was calculated for all compounds that lie in one of the 11 exemplar classes, in addition to the mean and variance for all compounds that do not. Assuming the mean and variance of the log transformed maximum similarities for classified compounds are *μ*_*c*_ and $\sigma_{c}{}^{2}$, and the corresponding measures for unclassified compounds are *μ*_*u*_ and $\sigma_{u}{}^{2}$, and the prior probability that a compound is classified is *P*_*c*_, then according to Bayes’ rule, the log transform of the maximum similarity threshold is:

$$m_{t}=\frac{\sigma_{c}{}^{2}\mu_{u}-\sigma_{u}{}^{2}\mu_{c}+\sigma_{c}\sigma_{u}\sqrt{{\left(\mu_{c}-\mu_{u}\right)}^{2}+\left(\sigma_{c}{}^{2}-\sigma_{u}{}^{2}\right)\text{log}\frac{\sigma_{c}{}^{2}}{\sigma_{u}{}^{2}}\frac{P_{c}}{1-P_{c}}}}{\left(\sigma_{c}{}^{2}-\sigma_{u}{}^{2}\right)}$$

Passing this value back through an exponential transform gives us the classification threshold we seek. Note that when *P*_*c*_ is assumed to be 0.5 (that is, it is equally likely that the compound is classified or unclassified), the equation above simplifies to the much more elegant form:

$$m_{t}=\frac{\sigma_{c}\mu_{u}+\sigma_{u}\mu_{c}}{\sigma_{c}+\sigma_{u}}$$

However, for our classifications, we elected to use the proportion of training compounds that belonged to one of the 11 exemplar classes (≈0.68). Those compounds that exceeded the threshold were assigned to the mechanism class with which they achieved the highest similarity, and the remaining compounds were designated “Unspecified”. This is reported as the “Predicted Class”.

## Results

### Advantages of LDA vs. PCA

Simply put, PCA finds dimensions that best explain the variability within a data set, whereas LDA finds the dimensions that best discriminate the classes embedded in the data. In this study, we expanded the concept of “class” to include each concentration tested for every compound. With M compounds tested at N concentration points, we trained a multi-class LDA model to discriminate between MxN classes. This approach finds dimensions in the data that best discriminate by both compound and concentration, enabling the identification of common phenotypic trajectories shared by mechanistically-similar compounds.

**Fig 2:**
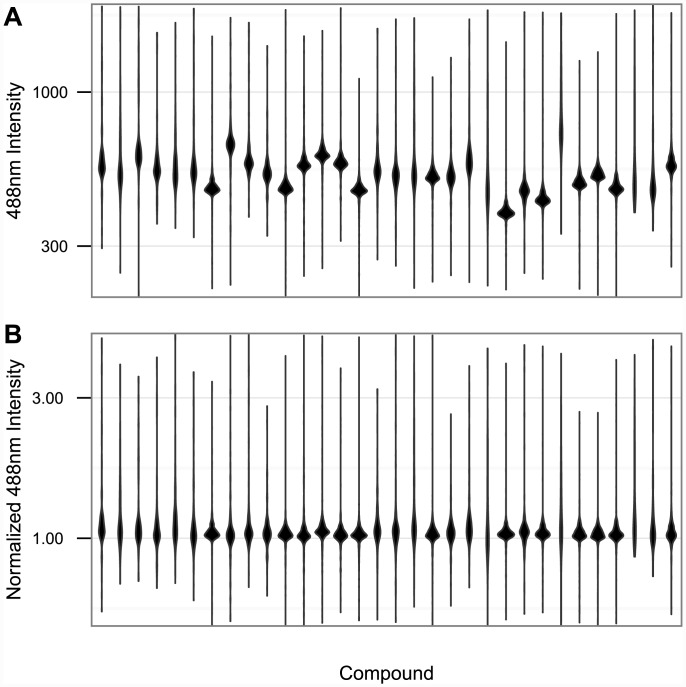
p-H2A.X Stain Normalization. **(A)** Violin plot depicting the distributions of cytoplasmic p-H2A.X stain intensity across all cells in 32 wells containing 32 different compounds at the highest concentrations used in analysis. The distributions show high levels of inter-compound variation, suggesting the dimension could be used to distinguish compounds. **(B)** Violin plot depicting the distributions of the same measurements as described in (A) normalized to the background level of 488nm intensity in each well. While some small differences remain, almost all inter-well variation has been eliminated.

It was interesting, then, that one of the earliest benefits of using multiclass LDA came not in the form of improved accuracy in mechanism clustering, but in the identification of a previously unknown source of experimental noise. When LDA was first run on the set of 154 training compounds, the three most putatively informative dimensions consistently contained a dimension of variability that seemed to carry very little information at all. Though the dimension displayed a great deal of variation from one compound to the next, it also displayed great variability from one dose of a given compound to the next, with little to no discernible pattern. Visual evaluation and manual rotation allowed us to determine that the dimension was dominated by the average level of phosphorylated histone H2A family member X (p-H2A.X) staining both inside and outside the nucleus. This led us to examine the distributions of 488nm intensities (which represent the levels of p-H2A.X [Ser139]) in the original images. As Fig 2A shows, the level of 488nm intensity exhibited a great deal of variability from one well to another, leading to a correspondingly high level of variation from one compound to another in our LDA. This turned out to be a result of a variation in overall 488nm intensity from one image to the next, and not variability produced by the underlying compounds. Normalization of the background 488nm intensity of each image all but eliminated this variability (Fig 2B). This insight would have been difficult to achieve using PCA, because the magnitude of p-H2A.X variability was small relative to the total variation in the data set, yet this noise happened to be a significant (and erroneous) means of discriminating between compounds.

**Fig 3:**
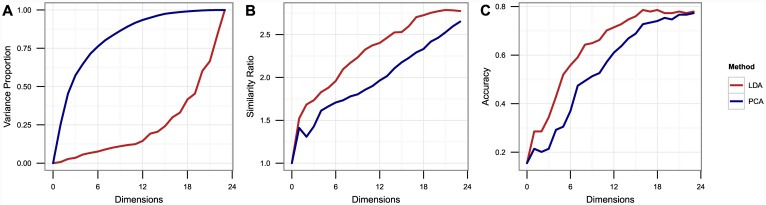
Performance of LDA vs. PCA. **(A)** Proportion of total variance accounted for in the first *n* dimensions as extracted by PCA and LDA. **(B)** Ratio of average MSWO similarity between compounds in the same mechanism class to average similarity between compounds in different classes using the first *n* dimensions extracted by PCA and LDA. **(C)** Classification accuracy achieved using our classification algorithm and the first *n* dimensions extracted by PCA and LDA.

The strength of LDA, of course, was not limited to elimination of experimental noise. Fig 3 depicts a comparison of reduced dimensionality subspaces calculated using PCA and multi-class LDA across several metrics of representational efficacy. The first, and most common metric, is the proportion of overall variance accounted for by a given *n*-dimensional subspace. PCA handily outperforms LDA on this scale, which is hardly surprising as it is this objective which PCA is specifically designed to optimize. Fig 3B and 3C, however, depict two metrics more appropriate to the task at hand. The first is a ratio of average intra-class similarity (calculated using maximum sequential weighted overlap, or MSWO, see Methods) to average interclass similarity. While increasing dimensionality improves this metric for both methods, LDA provides a consistently higher ratio, with an additional three or four PCA dimensions needed to reach the same level of discriminability. The third and final metric is the accuracy of our classification algorithm (see Methods for full details). As with the similarity ratio, LDA subspaces consistently outperform PCA subspaces, with PCA needing an extra three or four dimensions to achieve similar performance. Based on the flat behavior in the first three or four PCA subspaces, this finding is likely because the first several PCA dimensions correspond to high global variability, but little inter-compound variation, and thus contributes little to the recognition or identification of compound phenotype. This plot also shows that maximum performance is achieved using the first 16 LDA dimensions, suggesting that 16 of the 23 dimensions constitute the optimal bias-variance tradeoff for mechanistic classification. Unless otherwise specified, we use these 16 dimensions in all subsequent analyses.

### Identified Phenotypic Tracks

**Fig 4:**
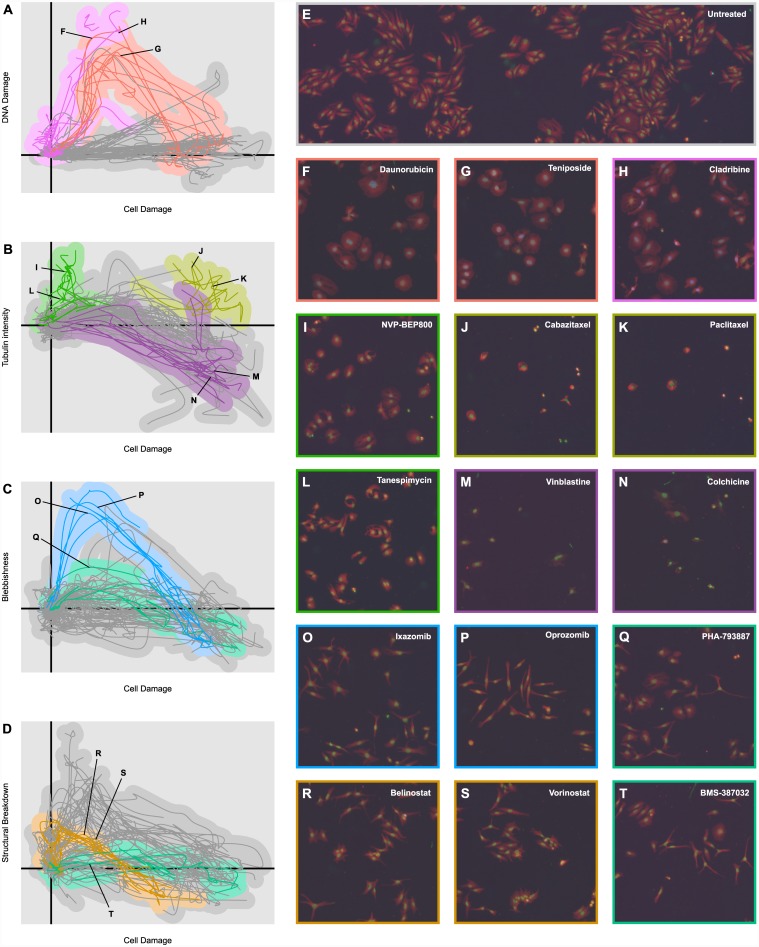
Example Phenotypic Tracks. **(A)** Plot of the means of the phenotypic trajectories of all 154 compounds in two highly informative dimensions, “Cell Damage” and “DNA Damage”. Topoisomerase inhibitors (red) and antimetabolites (magenta) have been highlighted. **(B)** Plot of phenotypic trajectory means in the “Cell Damage” and “Tubulin Intensity” dimensions. Microtubule stabilizers (yellow), microtubule inhibitors (purple), and HSP90 inhibitors (green) have been highlighted. **(C)** Plot of phenotypic trajectory means in the “Cell Damage” and “Blebbishness” dimensions. Proteasome inhibitors (blue) and CDK inhibitors (teal) have been highlighted. **(D)** Plot of phenotypic trajectory means in the “Cell Damage” and “Structural Damage” dimensions. HDAC inhibitors (dark orange) and CDK inhibitors (teal) have been highlighted. **(E)** Example microscopy image of untreated cells. The red channel depicts α-tubulin intensity, the green channel Hoescht stain intensity, and the blue channel p-H2A.X stain intensity. **(F–T)** Example microscopy images of cells treated by 15 different compounds from the mechanism classes highlighted in Fig 4A–4D. The points in the phenotypic trajectories to which the images correspond have been indicated where appropriate.

Visual examination of the most informative dimensions reveals several clearly discernible phenotypic tracks dominated by the trajectories of one or a small number of compound mechanism classes. Furthermore, manual rotation and inspection of these dimensions separates many of these tracks into distinct phenotypic dimensions associated with easily understood cellular properties. Here, we discuss several such phenotypic tracks (depicted in Fig 4).

### The DNA Damage Track

Fig 4A plots the phenotypic trajectories of all 154 compounds on two highly informative phenotypic dimensions. The *x*-axis, described as “Cell Damage” is one of the more complex dimensions, but is largely characterized by a decrease in cell size, an increase in cell roundness, an increase in nuclear DNA content, and a small increase in p-H2A.X staining. Nearly all compounds that reduce cell count compared to negative controls move positively along this dimension, and the relationship between variation on this dimension and cell count is tight enough that plotting other phenotypic dimensions against the “Cell Damage” dimension gives an intuitive representation of how cell phenotype changes as drugs become more cytotoxic.

The second dimension, referred to as “DNA Damage” is, unsurprisingly, primarily driven by intensity of p-H2A.X staining inside the nucleus. As the plot shows, while most drugs are relatively unperturbed in this dimension, two mechanism classes—topoisomerase inhibitors and antimetabolites—show a large increase compared with untreated cells. In addition, the topoisomerase inhibitors return to the lower values in this dimension at higher, more cytotoxic concentrations; as a result, the phenotypic distributions for the highest concentrations of some topoisomerase inhibitors are difficult to distinguish from those of many other drugs, making the use of dose response behavior particularly valuable for this class. Examples of cells corresponding to this phenotypic track are shown in Fig 4F–4H.

#### The Microtubule Track

Fig 4B plots the “Cell Damage” dimension against another highly informative and biologically intuitive dimension: the “Tubulin Intensity” dimension is governed almost entirely by the intensity of α-tubulin intensity both inside and outside the nucleus. As expected, microtubule stabilizers like the taxanes show an elevated response along this dimension compared with other similarly potent treatments, while the microtubule inhibitors show a relatively lower level throughout their phenotypic trajectories. Another mechanism class, however, distinguishes itself along this dimension: the heat shock protein 90 (HSP90) inhibitors, which show elevated levels of tubulin intensity coupled with a relatively low level of cell damage. In fact, the HSP90 inhibitors produce a very tight phenotypic cluster that exhibits idiosyncratic behavior along a number of phenotypic dimensions, but this is one of the most pronounced. Examples of cells in each of these phenotypic tracks are shown in Fig 4I–4N.

#### The Blebbish Track

Fig 4C and 4D plot the “Cell Damage” dimension against two more highly informative dimensions, referred to as the “Blebbishness” and “Structural Damage” dimensions. Mathematically, the “Blebbish” dimension is represented by a delicately balanced set of morphological parameters, including an increase in cell radius relative to cell area, an increase in eccentricity, and a decrease in compactness (neither “blebbishness” nor anything similar was explicitly measured in the image processing software), while the “Structural Damage” dimension describes the spatial relationships between the distribution of stains and cellular compartments, factoring in α-tubulin staining outside the cell, DNA staining outside the nucleus, and increased tetraploidy. The “Blebbish” dimension is particularly interesting because it makes little to no appearance in the first ten dimensions extracted by PCA, because the variations that define it, though statistically significant, are small compared with overall variations. One compound class known to induce blebbing, the proteasome inhibitors, [17] shows a pronounced and unmistakable deviation in this dimension (though, as with the topoisomerase inhibitors, only at middling to lower concentrations). Visual examination of the cells exposed to these treatments reveals that they do indeed exhibit a blebbing phenotype.

The proteasome inhibitors, however, are not the only treatments that induce a blebbing appearance. The HDAC inhibitors and some cyclin-dependent kinase (CDK) inhibitors produce visually similar phenotypes; but these mechanism classes produce only small deviations on the morphology-based “Blebbishness” dimension. This suggests that the “Blebbishness” dimension captures only one facet of the blebbing phenotype, a facet which is most strongly present in the proteasome inhibitors. It also demonstrates how the human limitations of visual microscopy can be complemented by the strengths of quantitative analysis: though the phenotypes of the proteasome inhibitors, CDK inhibitors, and HDAC inhibitors are visually similar (Fig 4O–4T), the plots of their phenotypic trajectories in Fig 4C and 4D show that the phenotypes are quantitatively highly distinct. This impression is confirmed by the clustering and classification results below, in which the three mechanism classes are robustly separated.

### Hierarchical Clustering and Classification

#### Training Compounds

**Fig 5:**
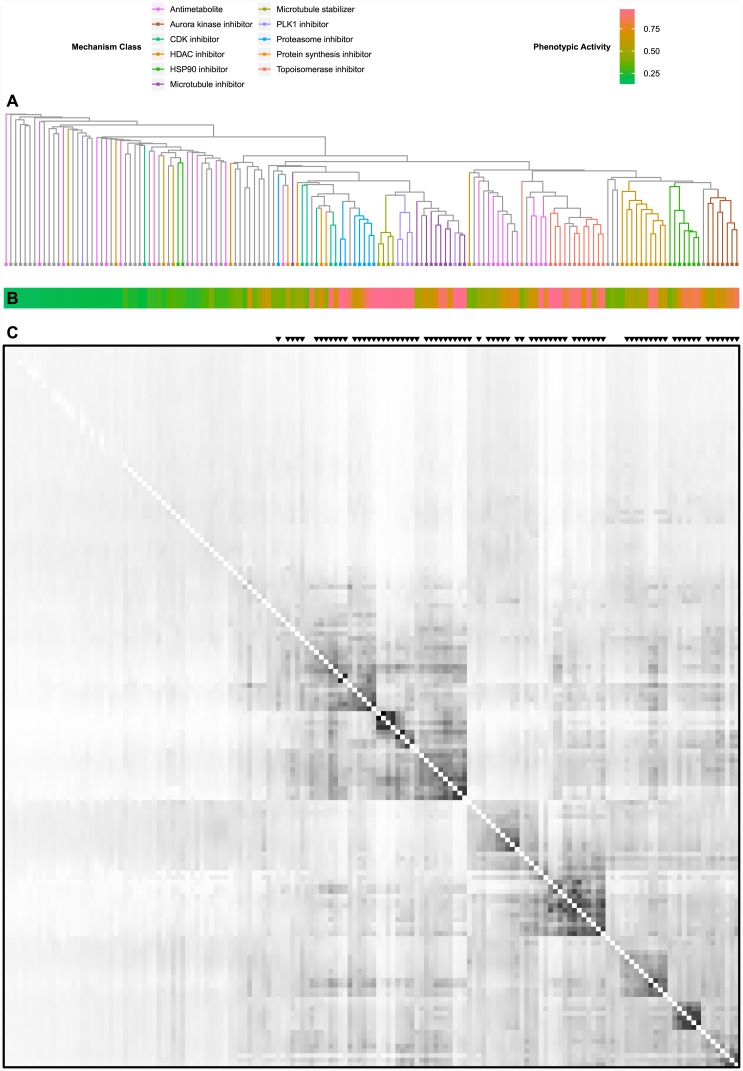
Hierarchical Compound Clustering. **(A)** Dendrogram of similarity-based hierarchical clustering. For more detail, see S1 Fig. **(B)** Heatmap depicting phenotypic activity of all compounds in the dendrogram in Fig 5A. **(C)** Similarity matrix for all 154 training compounds (white indicates the minimum similarity of 0, black indicates the maximum similarity of 1).

A hierarchical clustering of the 154 training compounds resulting from the MSWO similarity measure is shown in Fig 5 (a full copy of this clustering tree, with compound labels, can be found in S1 Fig). Compounds belonging to the 11 exemplar classes used in the classification are highlighted (a full listing of the phenotypic activities of all 154 compounds and how they were classified can be found in S3 Table).The heat map of phenotypic activity (Fig 5B) beneath the tree demonstrates that the ability of compounds to form similarity clusters is tightly correlated with phenotypic activity. From the similarity matrix below (Fig 5C), it is easy to see why: compounds with low phenotypic activities also show low similarity scores with other compounds across the board.

Of the 154 training compounds, classification using a leave-one-out cross-validation correctly classified 120 compounds for a hit rate of 78% (chance performance being 16%). Performance improved when less phenotypically active compounds were excluded: compounds with a phenotypic activity greater than 0.3 (111 compounds) were correctly classified at a rate of 80%, and compounds with a phenotypic activity greater than 0.5 (80 compounds) were correctly classified at a rate of 89%. Most encouragingly, compounds belonging to one of the 11 exemplar classes with a phenotypic activity greater than 0.3 (87 compounds) were correctly classified at a rate of 90%. All aurora kinase inhibitors, microtubule inhibitors, microtubule stabilizers, proteasome inhibitors, and topoisomerase inhibitors were classified correctly. The 10 most phenotypically active compounds that were incorrectly classified are shown in Table 1.

**Table 1:** 10 most phenotypically active misclassified compounds.

Drug	Expected Class	PA	Best Match Class	Predicted Class
Gemcitabine	Antimetabolite	0.980	Topoisomerase inhibitor	Unspecified
Rigosertib	PLK1 inhibitor	0.980	Microtubule inhibitor	Microtubule inhibitor
Mitomycin	Unspecified	0.887	Topoisomerase inhibitor	Topoisomerase inhibitor
Thapsigargin	Unspecified	0.854	Proteasome inhibitor	Proteasome inhibitor
Cladribine	Antimetabolite	0.767	Topoisomerase inhibitor	Topoisomerase inhibitor
FdCyd	Unspecified	0.754	Antimetabolite	Antimetabolite
Cytarabine	Antimetabolite	0.641	Topoisomerase inhibitor	Topoisomerase inhibitor
Rotenone	Unspecified	0.599	Microtubule inhibitor	Microtubule inhibitor
Bleomycin sulfate	Unspecified	0.514	Topoisomerase inhibitor	Topoisomerase inhibitor
Thioguanine	Antimetabolite	0.479	HDAC inhibitor	Unspecified

#### Model Comparison

**Fig 6:**
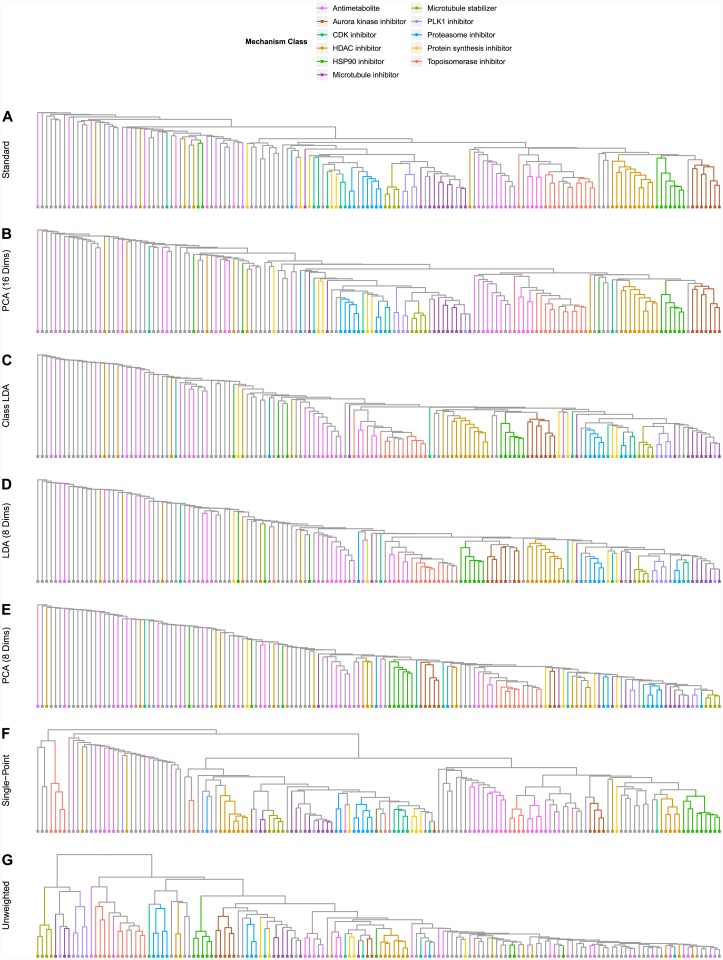
Clustering in Alternate Models. **(A)** Hierarchical clustering resulting from our complete model. **(B)** Clustering resulting from using the 16 best PCA dimensions, rather than the 16 best LDA dimensions. **(C)** Clustering resulting from 11 best dimensions extracted through mechanism class-based LDA. **(D)** Clustering resulting from using only 8 LDA dimensions. **(E)** Clustering resulting from using only 8 PCA dimensions. **(F)** Clustering resulting from using only the highest concentration of each compound (no dose response). **(G)** Clustering resulting from using simple overlap in the MSWO calculation rather than weighted overlap.

**Fig 7:**
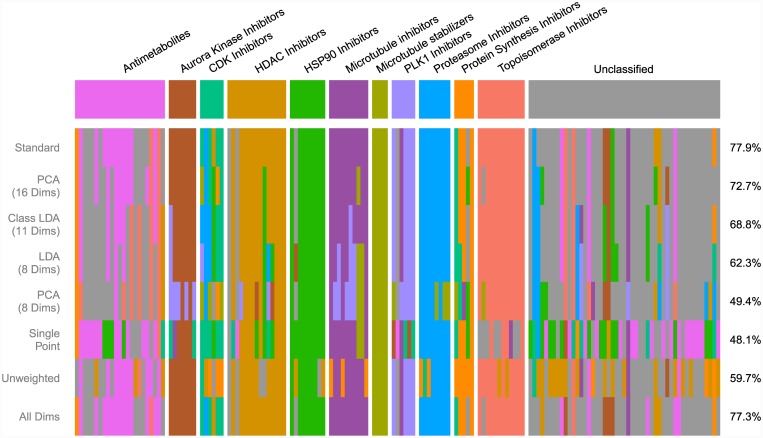
Classification in Alternate Models. Breakdown of classification performance of alternate analysis models by mechanism class. “All Dims” depicts classification performance when no dimensional reduction is performed prior to classification.

Our results suggest that phenotypic analysis and classification is most effective when dimensions extracted through treatment-based LDA are used on dose response data with a weighted similarity metric that attenuates similarity between phenotypically inactive treatments. Fig 6 depicts the clustering results of our model in comparison with several alternative models which lack at least one of these critical features; Fig 7 depicts the classification results of each of these alternate models broken down by compound mechanism. Use of PCA rather than LDA introduces subtle degradations in the clustering and classification performance; the effect is even more pronounced when a minority of extracted dimensions is used. Here, using PCA rather than LDA causes the aurora kinase inhibitors, HDAC inhibitors, and proteasome inhibitors to fail to form distinct clusters. Use of mechanism class-based LDA limits the number of extracted dimensions and degrades handling of complex multi-cluster classes such as the antimetabolites Use of single-point measurements and un-weighted similarity scores causes even larger drops in performance, particularly in reliable identification of compounds that do not belong to one of the exemplar classes.

#### Test Compounds

**Fig 8:**
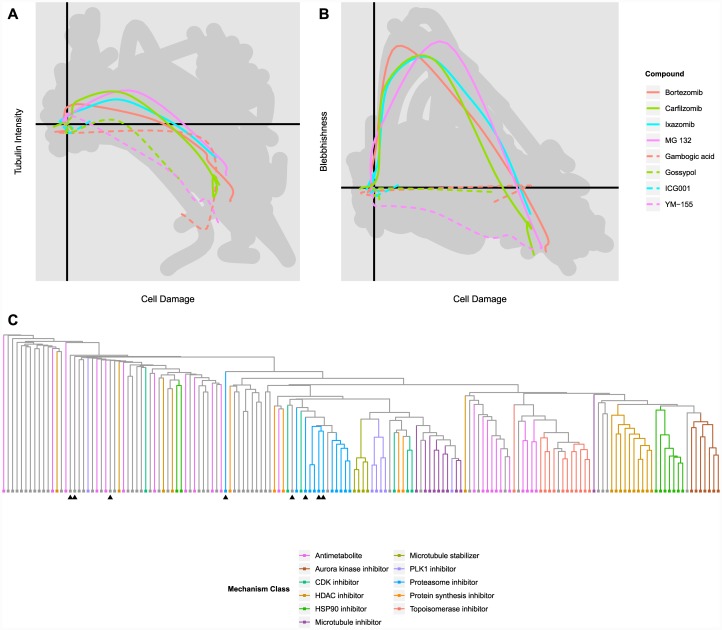
Phenotypic Analysis of Test Compounds. **(A)** Plot of phenotypic trajectory means for all eight test compounds in the “Cell Damage” and “Tubulin Intensity” dimensions. The extent of phenotypic trajectory means in the 154 test compounds has been included in grey for reference. Proteasome inhibitors are indicated by solid lines; unclassified compounds are indicated by dotted lines. **(B)** Plot of phenotypic trajectory means for all eight test compounds in the “Cell Damage” and “Blebbishness” dimensions. **(C)** The complete hierarchical clustering resulting from including the eight test compounds in the set. The eight test compounds have been indicated with black triangles. For more detail, see S2 Fig.

To assess reproducibility and predictive power, we applied our clustering and classification method to 8 compounds from a drug plate that was not part of the original training set. The plate contained four proteasome inhibitors, all of which appeared in the training compounds, and four compounds that were not reported to belong to any of the exemplar classes. Using the dimensions extracted from the multi-class LDA run on the 154 training compounds, plots of the 8 novel trajectories on the “Cell Damage”, “Tubulin Intensity”, and “Blebbishness” dimensions are shown in Fig 8A and 8B. Though all eight test compounds follow standard trajectories in the “Cell Damage” and “Tubulin Intensity” dimensions, the four proteasome inhibitors exhibit the same increasing and decreasing phenotypic trajectory in the “Blebbishness” dimension as was exhibited by their counterparts in the training set.

Again using the 16 most informative phenotypic dimensions extracted from the training compounds, similarity between the eight test compounds and the training compounds was estimated. The results of the expanded hierarchical clustering are shown in Fig 8C (a more complete version of this clustering tree, with compounds labeled, can be found in S2 Fig); as expected, three of the four proteasome inhibitors cluster tightly with the proteasome inhibitors in the training compounds, while a fourth (MG 132) exhibited a low phenotypic activity and thus achieved corresponding low similarity scores. Of the four unknown compounds, three exhibited low phenotypic activities (<0.3); but the fourth, the survivin inhibitor YM-155, showed a significantly higher activity measure, and clustered near the proteasome inhibitors. This clustering, however, was not robust: when the analysis was performed using 15 or 17 dimensions, YM-155 clustered differently. This behavior made YM-155 similar to other highly phenotypically active but idiosyncratic compounds such as ouabain, auranofin, and staurosporine, which were differently misclassified based on small changes in the conditions of the analysis.

Finally, the results of the similarity-based compound classification are shown in Table 2. All four proteasome inhibitors were correctly classified, even phenotypically weak MG 132. Of the four unknown compounds, the three phenotypically inactive compounds were correctly grouped into the ‘Unspecified’ class, while YM-155 was incorrectly classified as a CDK inhibitor. Interestingly, recent reports suggest that YM-155 might exert pleotropic effects beyond the inhibition of survivin [18] For example, YM-155 can block the association of the transcription factor SP1 to promoter sites, which decreases the expression of cyclin D1 and induces a cell-cycle arrest phenotype [19] that could also be comparable to that achieved by pan-CDK inhibition.

**Table 2:** Classification results for all eight test compounds.

Drug	Expected Class	PA	Best Match	Predicted Class
Bortezomib	Proteasome inhibitor	0.588	Proteasome inhibitor	Proteasome inhibitor
Carfilzomib	Proteasome inhibitor	0.544	Proteasome inhibitor	Proteasome inhibitor
Ixazomib	Proteasome inhibitor	0.421	Proteasome inhibitor	Proteasome inhibitor
MG 132	Proteasome inhibitor	0.273	Proteasome inhibitor	Proteasome inhibitor
YM-155	Unspecified	0.498	CDK inhibitor	CDK inhibitor
Gambogic acid	Unspecified	0.277	Protein synthesis inhibitor	Unspecified
ICG001	Unspecified	0.214	Antimetabolite	Unspecified
Gossypol	Unspecified	0.204	HDAC inhibitor	Unspecified

Thus, although YM-155 is considered a classification error, our method provided potentially important insight into its mechanism of action. This finding suggested that we should more closely examine the behavior of outliers from our training set.

### Selected Classification Outliers

#### Antimetabolites

When originally assigning mechanism classes, classification of the antimetabolites was a problematic decision. Though they constituted the largest class (23 of the 154 test compounds), and subdivisions of antimetabolites certainly do exist, these subdivisions are multifaceted and overlapping, making the selection of a single rational partition of the class difficult. To avoid biasing results with a largely subjective decision, the antimetabolites were left as a single monolithic class. Yet the hierarchical clustering results from Fig 5 clearly show that among the more phenotypically active antimetabolites, two distinct phenotypic clusters exist: one closely grouped with the topoisomerase inhibitors, and another forming an adjacent but distinct compact cluster. These clusters are not merely an arbitrary division resulting from experimental noise: the compounds comprising these clusters target distinct metabolic mechanisms. The first cluster, which exhibits high deviation along the “DNA Damage” dimension, much like the topoisomerase inhibitors, consists of the DNA polymerase inhibitors cytarabine and its pro-drug ancitabine hydrochloride; and the ribonucleotide reductase inhibitors gemcitabine and cladribine, all of which directly inhibit the repair of DNA damage. The second cluster, which shows a significantly smaller deviation on the “DNA Damage” dimension, includes floxuridine, pralatrexate, raltitrexed, methotrexate, trifluridine, and pemetrexed, all antifolates that have multiple effects including the inhibition of thymidylate synthase (TS), which eventually results in DNA damage. This distinction, though biologically relevant, is not one which was known to be phenotypically relevant prior to our analysis. The use of this assay, therefore, has allowed us to develop a more nuanced understanding of the phenotypic behavior of antimetabolites, which can improve our analysis and identification of such compounds in future work.

#### FdCyd

5-Fluoro-2’-Deoxycytidine (FdCyd) is a pyrimidine analog currently in clinical trials as a DNA methyltransferase inhibitor.[20,21] Though other DNA methyltransferase inhibitors were included in the test set of drugs, including azacitidine, decitabine, and zebularine, FdCyd unexpectedly grouped more strongly with the phenotypically active antifolates discussed in the previous section; in fact, the similarity between FdCyd and floxuridine was greater than the similarity between any pair of antifolates. Further review of the literature revealed that FdCyd is administered in conjunction with tetrahydrouridine (THU), which inhibits the action of cytidine deaminase and prevents the metabolism of FdCyd into 5-fluoro-2’deoxyuridine—a potent inhibitor of TS.[22] Thus, in the presence of cytidine deaminase, FdCyd can, in addition to its action as a DNA methyltransferase inhibitor, behave like a potent TS inhibitor. Cytidine deaminase levels wary widely in human tumors [23] and can be augmented by cell culture conditions.[24] As THU was not applied in our minimalist assay, and given uncertain cytidine deaminase expression, the classification of FdCyd as an antimetabolite and the clustering of FdCyd with the TS-inhibiting antifolates does not represent a failure of the phenotypic assay. Rather, this indicates an accurate identification of an off-target effect that had not yet been accounted for by our analysis.

#### Rigosertib

Another compound that grouped very strongly with a class other than its published mechanism class is ON01910.Na, or rigosertib. Currently in Phase II clinical trials for the treatment of myelodysplactic syndromes, rigosertib was originally reported, and is generally listed, as a polo-like kinase 1 (PLK1) inhibitor.[25] Hierarchical clustering and similarity-based classification in our assay, however, suggested that rigosertib was acting not as a PLK1 inhibitor, but as a microtubule inhibitor or disruptor. Subsequent research on rigosertib has shown that while it can phenocopy the behavior of a PLK1 inhibitor, it does not directly inhibit PLK1.[26] Recent analysis of a close analog, TL-77, revealed that both drugs inhibit tubulin polymerization, interfere with mitotic spindle assembly, and may indirectly suppress the PLK1 pathway.[27] Thus, as with FdCyd, what appears to be a misclassification instead represents the identification of an unanticipated off-target effect—precisely the kind of effect such an assay should identify.

## Discussion

We have described a new analytical paradigm for interpreting high content phenotypic screening experiments. The use of multi-class treatment-based LDA rather than more traditional factor analysis such as PCA enabled the identification of assay artifacts while improving clustering and classification accuracy. Visual inspection and manual rotation of the most informative LDA dimensions allowed us to identify biologically intuitive and highly informative phenotypic dimensions that successfully discriminated several mechanistic classes. The flat accuracy curve displayed by PCA subspaces in Fig 3C demonstrates that several of the initial phenotypic dimensions extracted by PCA carry little information that distinguishes one compound from another; therefore visual examination of these dimension would offer little to no insight about what defines a given mechanism class’s particular phenotypic behavior. Furthermore, as additional stains and more complex or targeted cellular measurements are added to an assay, the total dimensionality of the data can easily grow faster than the space of informative dimensions. Hence, robust identification of the most informative dimensions through treatment-based LDA will become increasingly essential as our approach is extended to less minimalist assays. Finally, the most informative dimensions produced by LDA, because they consists of linear combinations of phenotypic measurements, can be easily interpreted in terms of phenotypic measurements, making their biological significance more accessible than representations extracted by non-linear approaches like multidimensional analysis or self-organizing maps.

The use of dose-response data, though it requires a greater investment of time and material, yielded a significant improvement in the ability of our assay to identify phenotypic clusters and correctly classify phenotypically active compounds. Further, it revealed complex, multi-stage phenotypic tracks which are traversed by similarly acting compounds across a wide range of concentrations. Finally, we developed an inter-compound similarity metric (MSWO) that integrates information across doses and attenuates similarity between ineffective treatments, allowed for robust measurement of within-class similarity, and greatly improved classification accuracy and clustering.

Perhaps the most intriguing insight resulting from this study is the complexity and nuance that it revealed in the behavior of compounds within the same mechanistic class. Though the assay was originally developed to assist in the classification of novel or unknown molecules according to established mechanistic classes, several of the most interesting results have been those that reveal unexpected off-target effects, as in the case of two advanced clinical candidates—FdCyd and rigosertib, and the phenotypic complexity of classes like the antimetabolites. This suggests that an equally important application of this assay will be the identification of compounds that behave differently from other members within the same mechanistic class.

Though the results described here are promising, several mechanistic classes, including the alkylating agents, apoptotic agents, mTOR inhibitors, and PI3K inhibitors, were left unspecified in classification because they contained too few sufficiently phenotypically active exemplar compounds. Lack of sufficient phenotypic activity is a significant limitation for high content phenotypic screening that has compelled the field to add more and more assays focused on specific molecular processes. However, our minimalist assay—measuring only DNA content, DNA damage, and tubulin—did remarkable well at discriminating several mechanistic classes. Additional dyes that capture orthogonal aspects of cell morphology, such as those staining the mitochondrial membrane, endoplasmic reticulum, or Golgi, would almost certainly increase the power of our analytical method to discern mechanistic behavior, and require only a nominal increase in time, effort, and cost.

We also recognize that time and the cellular environment play critical roles in determining a compound’s phenotypic response. Our assay was limited to a signal time point in a single cell line. One can imagine adding additional cell lines and time points, though at some point this will lead to diminishing returns. More importantly, we believe that our methodology is not ideal for determining the precise molecular mechanism of action, but rather is best used to identify phenotypic signatures that provide biologically-meaningful insight and a means to compare different compounds. Indeed, as was discussed earlier in the case of the antimetabolites, the definition of a mechanistic class is somewhat subjective and depends on the granularity you wish to impose. On the other hand, examining the morphological changes in a cell upon compound addition connects a molecular-scale event (e.g., the binding of an enzyme inhibitor) to a macroscopic effect and informs our understanding of the underlying biology.

The increasing accuracy and decreasing cost of automated microscopy have made high content screening and analysis an invaluable tool in the discovery and understanding of novel therapeutics. Yet the apparent promise of high content phenotypic screening still stands far from the reality of existing phenotypic analysis approaches. Given the variety of tools available and the increasing ease with which massive datasets may be collected, stored and accessed, it is tempting to address the limitations of high content analysis with ever more complex and data-rich assays, running several dozen stains and collecting hundreds of cellular measurements. The results presented here, however, show that careful analytical choices can yield varied and often unexpected insights in high content phenotypic assays, and that these insights do not require us to drown in ever-growing oceans of high content data.

## Supporting Information

S1 Fig Hierarchical clustering of all 154 training compounds. Similarity corresponds to average MSWO between clusters.
(PDF)

S2 Fig Hierarchical clustering of all 154 training compounds and 8 test compounds. Test compounds are marked by a double asterisk (**).
(PDF)

S1 Table 23 measurements used in analysis, and the transformation applied to them to produce a more normal distribution. (DOCX)

S2 Table List of all 154 training compounds, their published mechanism, the vendor that provided them, and the top concentration used in the assay. (DOCX)

S3 Table List of all training compounds, their phenotypic activities (PA), the mechanism used for the compound in classification, the best matching exemplar class, and the classification result. (DOCX)

S4 Table List of all 8 test compounds, their published mechanism, the vendor that provided them, and the top concentration used in the assay. (DOCX)
