# Supplementary figures and images for: Robust Classification of Small-Molecule Mechanism of Action Using a Minimalist High-Content Microscopy Screen and Multidimensional Phenotypic Trajectory Analysis
Source: PLoS One. 2016 Feb 17;11(2):e0149439. doi: 10.1371/journal.pone.0149439 (PMC4757101; doi:10.1371/journal.pone.0149439)

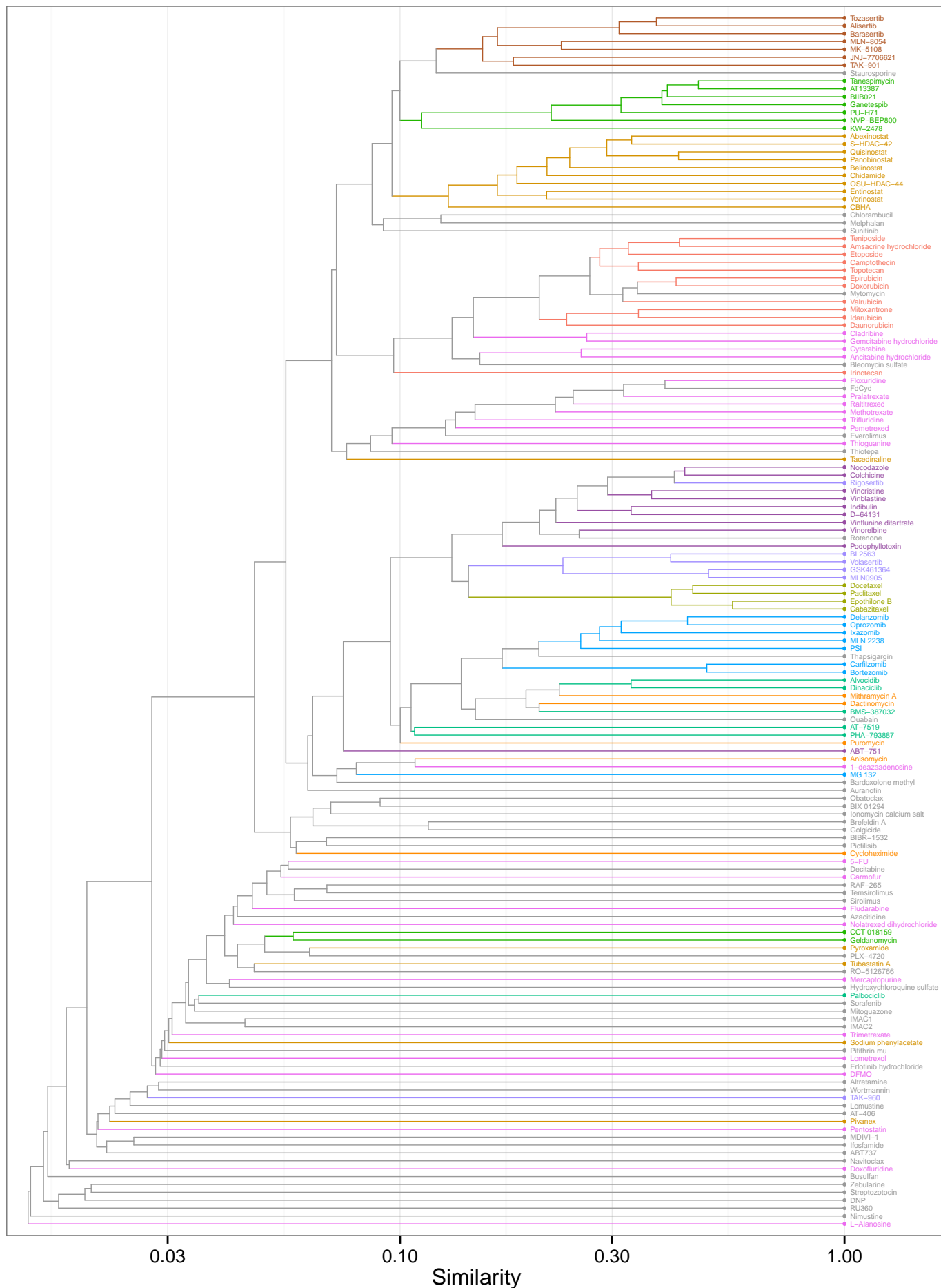

Supplement: S1 Fig — Similarity corresponds to average MSWO between clusters. (PDF) [file pone.0149439.s001.pdf]

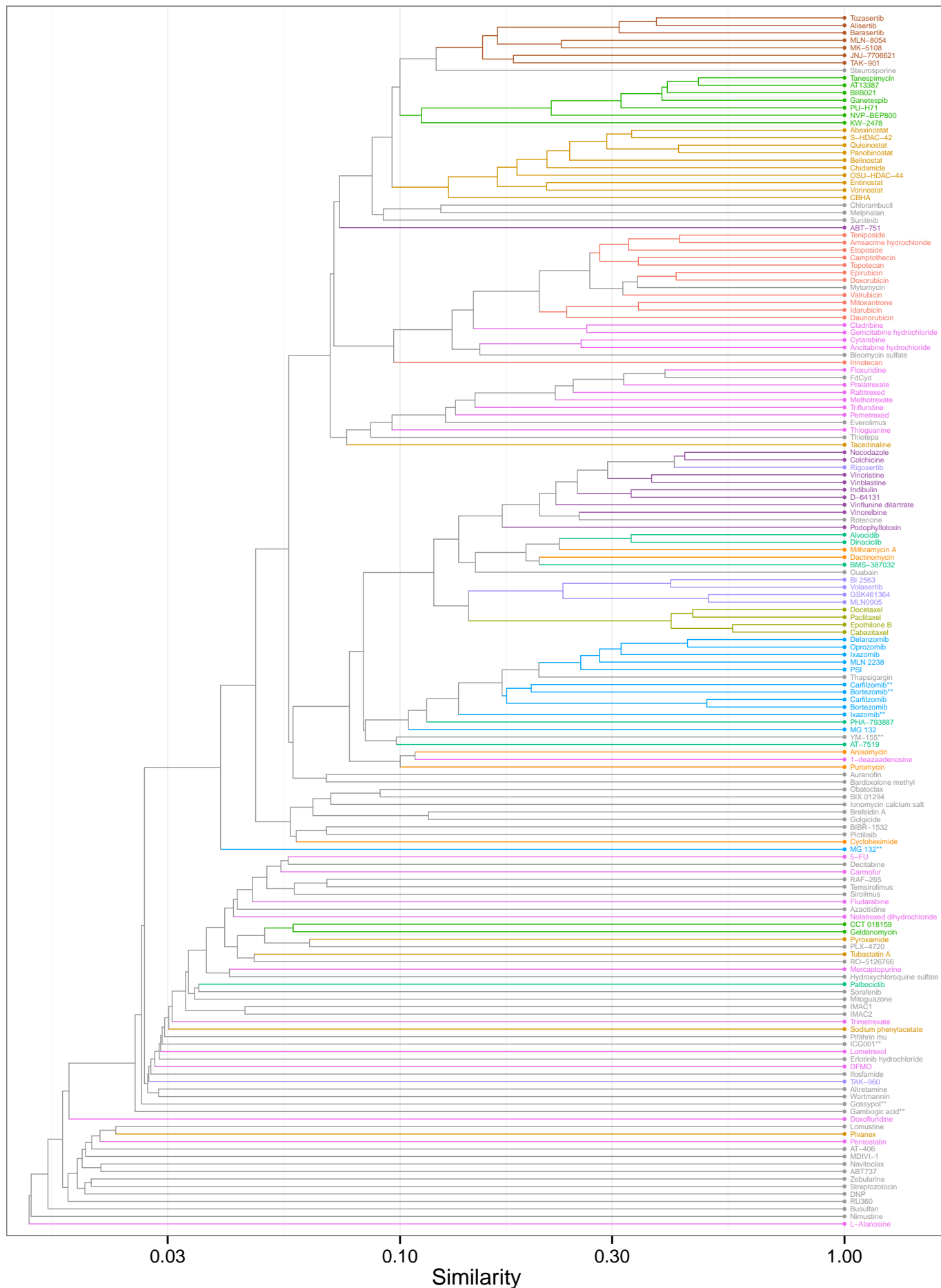

Supplement: S2 Fig — Test compounds are marked by a double asterisk (**). (PDF) [file pone.0149439.s002.pdf]
