# Supplementary material for: Robust Classification of Small-Molecule Mechanism of Action Using a Minimalist High-Content Microscopy Screen and Multidimensional Phenotypic Trajectory Analysis
Source: PLoS One. 2016 Feb 17;11(2):e0149439. doi: 10.1371/journal.pone.0149439 (PMC4757101; doi:10.1371/journal.pone.0149439)
Supplement: S1 Table — (DOCX) [file pone.0149439.s003.docx]

**S1 Table. 23 measurements used in analysis, and the transformation applied to them to produce a more normal distribution.**

| Measurement | Stain Used | Description | Transform |
| --- | --- | --- | --- |
| Nucleus Area | Hoescht | Area of the nucleus | Logarithm |
| Nucleus Gyration Radius* | Hoescht | Average radius of the nucleus | Logarithm |
| Nucleus Elongation | Hoescht | Mean ratio of the short axis of the nucleus to the long axis of the nucleus | Logit |
| Nucleus 1/(Form Factor) | Hoescht | Mean nucleus roundness index | Logarithm |
| Nucleus Displacement | Hoescht | Distance between the nucleus’s and the cell’s centers of gravity, normalized by the gyration radius of the nucleus | Logarithm |
| Nucleus Chord Ratio | Hoescht | Shortest chord to longest chord ratio. Both chords pass through the shape’s center of gravity | Logit |
| Cell Area | α-Tubulin | Area of the cell | Logarithm |
| Cell Gyration Radius | α-Tubulin | Average radius of the cell | Logarithm |
| Cell Elongation | α-Tubulin | Mean ratio of the short axis of the cell to the long axis of the cell | Logit |
| Cell 1/(Form Factor) | α-Tubulin | Mean cell roundness index | Logarithm |
| Nucleus Hoescht Intensity | Hoescht | Mean intensity of Hoescht stain in the nucleus | Logarithm |
| Nucleus Hoescht Intensity CV | Hoescht | Coefficient of variation of Hoescht stain intensity in the nucleus | Logarithm |
| Nucleus α-Tubulin Intensity | α-Tubulin | Mean intensity of α-Tubulin stain in the nucleus | Logarithm |
| Nucleus α-Tubulin Intensity CV | α-Tubulin | Coefficient of variation of α-Tubulin stain intensity in the nucleus | Logarithm |
| Nucleus pH2AX Intensity | pH2AX | Mean intensity of pH2AX stain in the nucleus | Logarithm |
| Nucleus pH2AX Intensity CV | pH2AX | Coefficient of variation of pH2AX stain intensity in the nucleus | Logarithm |
| Cytoplasm Hoescht Intensity | Hoescht | Mean intensity of Hoescht stain in the cytoplasm | Logarithm |
| Cytoplasm Hoescht Intensity CV | Hoescht | Coefficient of variation of Hoescht stain intensity in the cytoplasm | Logarithm |
| Cytoplasm α-Tubulin Intensity | α-Tubulin | Mean intensity of α-Tubulin stain in the cytoplasm | Logarithm |
| Cytoplasm α-Tubulin Intensity CV | α-Tubulin | Coefficient of variation of α-Tubulin stain intensity in the cytoplasm | Logarithm |
| Cytoplasm pH2AX Intensity | pH2AX | Mean intensity of pH2AX stain in the cytoplasm | Logarithm |
| Cytoplasm pH2AX Intensity CV | pH2AX | Coefficient of variation of pH2AX stain intensity in the cytoplasm | Logarithm |
| 4N Filter | Hoescht | Binary filter indicating tetraploidy | None |

*The Multi-Target Analysis package in the GE InCell Analyzer Workstation extracts a measurement called “Compactness” for nuclei; the measure “Nucleus Gyration Radius” was calculated from this measure and “Nucleus Area” for better consistency between nuclear and cellular morphological measurements
