# Supplementary material for: Robust Classification of Small-Molecule Mechanism of Action Using a Minimalist High-Content Microscopy Screen and Multidimensional Phenotypic Trajectory Analysis
Source: PLoS One. 2016 Feb 17;11(2):e0149439. doi: 10.1371/journal.pone.0149439 (PMC4757101; doi:10.1371/journal.pone.0149439)
Supplement: S2 Table — (DOCX) [file pone.0149439.s004.docx]

**S2 Table. List of all 154 training compounds, their published mechanism, the vendor that provided them, and the top concentration used in the assay.**

| Drug | Published Mechanism | Vendor | Top Concentration (μM) |
| --- | --- | --- | --- |
| Altretamine | Alkylating agent | AK Scientific | 51 |
| Busulfan | Alkylating agent | Toronto Research Chemicals | 56 |
| Chlorambucil | Alkylating agent | Sigma-Aldrich | 40 |
| Ifosfamide | Alkylating agent | Sigma-Aldrich | 56 |
| Lomustine | Alkylating agent | Bosche Scientific | 56 |
| Melphalan | Alkylating agent | APAC Pharmaceutical | 54 |
| Mitoguazone | Alkylating agent | Chem Service | 48 |
| Mytomycin | Alkylating agent | Bosche Scientific | 41 |
| Nimustine | Alkylating agent | LKT Laboratories | 18 |
| Streptozotocin | Alkylating agent | Bosche Scientific | 20 |
| Thiotepa | Alkylating agent | Sigma-Aldrich | 43 |
| 1-deazaadenosine | Antimetabolite | Tocris Bioscience | 62 |
| 5-FU | Antimetabolite | Sigma-Aldrich | 53 |
| Ancitabine hydrochloride | Antimetabolite | Sigma-Aldrich | 47 |
| Carmofur | Antimetabolite | Santa Cruz Biotechnology | 58 |
| Cladribine | Antimetabolite | Toronto Research Chemicals | 54 |
| Cytarabine | Antimetabolite | Sigma-Aldrich | 53 |
| DFMO | Antimetabolite | Alexis Biochemicals | 50 |
| Doxofluridine | Antimetabolite | Cayman Chemical | 65 |
| Floxuridine | Antimetabolite | Toronto Research Chemicals | 64 |
| Fludarabine | Antimetabolite | Toronto Research Chemicals | 56 |
| Gemcitabine hydrochloride | Antimetabolite | LC Laboratories | 48 |
| L-Alanosine | Antimetabolite | Toronto Research Chemicals | 56 |
| Lometrexol | Antimetabolite | Sigma-Aldrich | 47 |
| Mercaptopurine | Antimetabolite | Toronto Research Chemicals | 63 |
| Methotrexate | Antimetabolite | Sigma-Aldrich | 57 |
| Nolatrexed dihydrochloride | Antimetabolite | Toronto Research Chemicals | 58 |
| Pemetrexed | Antimetabolite | LC Laboratories | 55 |
| Pentostatin | Antimetabolite | National Cancer Institute | 55 |
| Pralatrexate | Antimetabolite | Selleck Chemicals | 52 |
| Raltitrexed | Antimetabolite | Bosche Scientific | 65 |
| Thioguanine | Antimetabolite | Sigma-Aldrich | 55 |
| Trifluridine | Antimetabolite | AK Scientific | 58 |
| Trimetrexate | Antimetabolite | Tocris Bioscience | 56 |
| ABT737 | Apoptotic agent | Selleck Chemicals | 61 |
| AT-406 | Apoptotic agent | Selleck Chemicals | 65 |
| IMAC2 | Apoptotic agent | Calbiochem | 62 |
| Navitoclax | Apoptotic agent | Selleck Chemicals | 59 |
| Obatoclax | Apoptotic agent | Selleck Chemicals | 66 |
| Alisertib | Aurora kinase inhibitor | ChemieTek | 33 |
| Barasertib | Aurora kinase inhibitor | Selleck Chemicals | 49 |
| JNJ-7706621 | Aurora kinase inhibitor | Selleck Chemicals | 55 |
| MK-5108 | Aurora kinase inhibitor | Selleck Chemicals | 59 |
| MLN-8054 | Aurora kinase inhibitor | Selleck Chemicals | 56 |
| TAK-901 | Aurora kinase inhibitor | Selleck Chemicals | 41 |
| Tozasertib | Aurora kinase inhibitor | LC Laboratories | 68 |
| Alvocidib | CDK inhibitor | Selleck Chemicals | 52 |
| AT-7519 | CDK inhibitor | Selleck Chemicals | 57 |
| BMS-387032 | CDK inhibitor | Selleck Chemicals | 52 |
| Dinaciclib | CDK inhibitor | ChemieTek | 62 |
| Palbociclib | CDK inhibitor | ChemieTek | 60 |
| PHA-793887 | CDK inhibitor | Toronto Research Chemicals | 51 |
| Azacitidine | DNA methyltransferase inhibitor | AK Scientific | 59 |
| Decitabine | DNA methyltransferase inhibitor | Selleck Chemicals | 62 |
| FdCyd | DNA methyltransferase inhibitor | TCI Chemicals | 32 |
| Zebularine | DNA methyltransferase inhibitor | Calbiochem | 57 |
| Brefeldin A | Golgi inhibitor | LC Laboratories | 56 |
| Golgicide | Golgi inhibitor | Calbiochem | 78 |
| Abexinostat | HDAC inhibitor | Selleck Chemicals | 78 |
| Belinostat | HDAC inhibitor | Selleck Chemicals | 80 |
| CBHA | HDAC inhibitor | Cayman Chemical | 53 |
| Chidamide | HDAC inhibitor | Cayman Chemical | 44 |
| Entinostat | HDAC inhibitor | LC Laboratories | 76 |
| OSU-HDAC-44 | HDAC inhibitor | Calbiochem | 46 |
| Panobinostat | HDAC inhibitor | LC Laboratories | 50 |
| Pivanex | HDAC inhibitor | Sigma-Aldrich | 56 |
| Pyroxamide | HDAC inhibitor | Tocris Bioscience | 44 |
| Quisinostat | HDAC inhibitor | Selleck Chemicals | 54 |
| S-HDAC-42 | HDAC inhibitor | Apex Biologix | 47 |
| Sodium phenylacetate | HDAC inhibitor | Santa Cruz Biotechnology | 56 |
| Tacedinaline | HDAC inhibitor | LC Laboratories | 46 |
| Tubastatin A | HDAC inhibitor | ChemieTek | 51 |
| Vorinostat | HDAC inhibitor | LC Laboratories | 79 |
| AT13387 | HSP90 inhibitor | Selleck Chemicals | 54 |
| BIIB021 | HSP90 inhibitor | Selleck Chemicals | 61 |
| CCT 018159 | HSP90 inhibitor | Tocris Bioscience | 65 |
| Ganetespib | HSP90 inhibitor | MedKoo Biosciences | 70 |
| Geldanomycin | HSP90 inhibitor | LC Laboratories | 56 |
| KW-2478 | HSP90 inhibitor | Selleck Chemicals | 59 |
| NVP-BEP800 | HSP90 inhibitor | Selleck Chemicals | 26 |
| PU-H71 | HSP90 inhibitor | Santa Cruz Biotechnology | 45 |
| Tanespimycin | HSP90 inhibitor | LC Laboratories | 46 |
| ABT-751 | Microtubule inhibitor | Selleck Chemicals | 58 |
| Colchicine | Microtubule inhibitor | AG Chemicals | 66 |
| D-64131 | Microtubule inhibitor | Santa Cruz Biotechnology | 58 |
| Indibulin | Microtubule inhibitor | Santa Cruz Biotechnology | 51 |
| Nocodazole | Microtubule inhibitor | Sigma-Aldrich | 69 |
| Podophyllotoxin | Microtubule inhibitor | Tocris Bioscience | 56 |
| Vinblastine | Microtubule inhibitor | Tocris Bioscience | 49 |
| Vincristine | Microtubule inhibitor | International Laboratory | 39 |
| Vinflunine ditartrate | Microtubule inhibitor | Toronto Research Chemicals | 35 |
| Vinorelbine | Microtubule inhibitor | Santa Cruz Biotechnology | 56 |
| Cabazitaxel | Microtubule stabilizer | LC Laboratories | 53 |
| Docetaxel | Microtubule stabilizer | AK Scientific | 58 |
| Epothilone B | Microtubule stabilizer | LC Laboratories | 55 |
| Paclitaxel | Microtubule stabilizer | LC Laboratories | 63 |
| DNP | Mitochondrial interference | Santa Cruz Biotechnology | 55 |
| IMAC1 | Mitochondrial interference | Tocris Bioscience | 62 |
| Ionomycin calcium salt | Mitochondrial interference | Cayman Chemical | 56 |
| MDIVI-1 | Mitochondrial interference | Calbiochem | 56 |
| Pifithrin mu | Mitochondrial interference | Enzo Life Sciences | 67 |
| Rotenone | Mitochondrial interference | Sigma-Aldrich | 56 |
| RU360 | Mitochondrial interference | Calbiochem | 56 |
| Everolimus | mTOR inhibitor | International Laboratory | 59 |
| Sirolimus | mTOR inhibitor | LC Laboratories | 58 |
| Temsirolimus | mTOR inhibitor | LC Laboratories | 46 |
| Pictilisib | PI3K inhibitor | LC Laboratories | 47 |
| Wortmannin | PI3K inhibitor | Selleck Chemicals | 56 |
| BI 2563 | PLK1 inhibitor | Selleck Chemicals | 66 |
| GSK461364 | PLK1 inhibitor | APAC Pharmaceutical | 56 |
| MLN0905 | PLK1 inhibitor | Selleck Chemicals | 49 |
| Rigosertib | PLK1 inhibitor | Selleck Chemicals | 40 |
| TAK-960 | PLK1 inhibitor | Santa Cruz Biotechnology | 56 |
| Volasertib | PLK1 inhibitor | ChemieTek | 60 |
| Bortezomib | Proteasome inhibitor | LC Laboratories | 59 |
| Carfilzomib | Proteasome inhibitor | ChemieTek | 56 |
| Delanzomib | Proteasome inhibitor | Toronto Research Chemicals | 56 |
| Ixazomib | Proteasome inhibitor | Selleck Chemicals | 56 |
| MG 132 | Proteasome inhibitor | Santa Cruz Biotechnology | 53 |
| MLN 2238 | Proteasome inhibitor | Selleck Chemicals | 56 |
| Oprozomib | Proteasome inhibitor | ChemieTek | 59 |
| PSI | Proteasome inhibitor | Calbiochem | 14 |
| Anisomycin | Protein synthesis inhibitor | Calbiochem | 50 |
| Cycloheximide | Protein synthesis inhibitor | Acros Organics | 63 |
| Dactinomycin | Protein synthesis inhibitor | AG Chemicals | 9 |
| Mithramycin A | Protein synthesis inhibitor | Toronto Research Chemicals | 56 |
| Puromycin | Protein synthesis inhibitor | AG Chemicals | 54 |
| PLX-4720 | Raf inhibitor | Selleck Chemicals | 61 |
| RAF-265 | Raf inhibitor | Selleck Chemicals | 53 |
| Amsacrine hydrochloride | Topoisomerase inhibitor | International Laboratory | 43 |
| Camptothecin | Topoisomerase inhibitor | Sigma-Aldrich | 63 |
| Daunorubicin | Topoisomerase inhibitor | AK Scientific | 48 |
| Doxorubicin | Topoisomerase inhibitor | Selleck Chemicals | 40 |
| Epirubicin | Topoisomerase inhibitor | TSZ Chem | 51 |
| Etoposide | Topoisomerase inhibitor | MP Biomedicals | 56 |
| Idarubicin | Topoisomerase inhibitor | Sigma-Aldrich | 65 |
| Irinotecan | Topoisomerase inhibitor | TSZ Chem | 58 |
| Mitoxantrone | Topoisomerase inhibitor | AK Scientific | 54 |
| Teniposide | Topoisomerase inhibitor | 3B Pharmachem International | 56 |
| Topotecan | Topoisomerase inhibitor | Bosche Scientific | 50 |
| Valrubicin | Topoisomerase inhibitor | USP | 37 |
| Erlotinib hydrochloride | Tyrosine kinase inhibitor | Santa Cruz Biotechnology | 55 |
| Sorafenib | Tyrosine kinase inhibitor | LC Laboratories | 40 |
| Sunitinib | Tyrosine kinase inhibitor | Chempacific | 52 |
| Auranofin | Antirheumatic agent | Enzo Life Sciences | 56 |
| Bardoxolone methyl | NF-κB pathway inhibitor | Cayman Chemical | 53 |
| BIBR-1532 | Telomerase inhibitor | Tocris Bioscience | 74 |
| BIX 01294 | Histone methyltransferase inhibitor | Tocris Bioscience | 44 |
| Bleomycin sulfate | DNA damage | Alfa Aesar | 56 |
| Hydroxychloroquine sulfate | Autophagy inhibitor | TCI Chemicals | 8 |
| Ouabain | Na+/K+, -ATPase inhibitor | MP Biomedicals | 56 |
| RO-5126766 | MEK inhibitor | Active Biochem | 64 |
| Staurosporine | Non-specific kinase inhibitor | LC Laboratories | 52 |
| Thapsigargin | SERCA inhibitor | Cayman Chemical | 56 |
