# Supplementary material for: Robust Classification of Small-Molecule Mechanism of Action Using a Minimalist High-Content Microscopy Screen and Multidimensional Phenotypic Trajectory Analysis
Source: PLoS One. 2016 Feb 17;11(2):e0149439. doi: 10.1371/journal.pone.0149439 (PMC4757101; doi:10.1371/journal.pone.0149439)
Supplement: S3 Table — (DOCX) [file pone.0149439.s005.docx]

**S3 Table. List of all training compounds, their phenotypic activities (PA), the mechanism used for the compound in classification, the best matching exemplar class, and the classification result.**

| Drug | PA* | Classification Input | Best Match Mechanism | Predicted Class |
| --- | --- | --- | --- | --- |
| Gemcitabine hydrochloride | 0.98 | Antimetabolite | Topoisomerase inhibitor | Unspecified |
| Cladribine | 0.767 | Antimetabolite | Topoisomerase inhibitor | Topoisomerase inhibitor |
| Floxuridine | 0.738 | Antimetabolite | Antimetabolite | Antimetabolite |
| Pralatrexate | 0.679 | Antimetabolite | Antimetabolite | Antimetabolite |
| Ancitabine hydrochloride | 0.669 | Antimetabolite | Antimetabolite | Antimetabolite |
| Raltitrexed | 0.663 | Antimetabolite | Antimetabolite | Antimetabolite |
| Cytarabine | 0.641 | Antimetabolite | Topoisomerase inhibitor | Topoisomerase inhibitor |
| Pemetrexed | 0.516 | Antimetabolite | Antimetabolite | Antimetabolite |
| Methotrexate | 0.513 | Antimetabolite | Antimetabolite | Antimetabolite |
| Thioguanine | 0.479 | Antimetabolite | HDAC inhibitor | Unspecified |
| Trifluridine | 0.469 | Antimetabolite | Antimetabolite | Antimetabolite |
| 5-FU | 0.294 | Antimetabolite | Antimetabolite | Antimetabolite |
| Carmofur | 0.289 | Antimetabolite | Antimetabolite | Antimetabolite |
| Mercaptopurine | 0.258 | Antimetabolite | Antimetabolite | Unspecified |
| Fludarabine | 0.254 | Antimetabolite | Antimetabolite | Antimetabolite |
| Nolatrexed dihydrochloride | 0.253 | Antimetabolite | Antimetabolite | Antimetabolite |
| Azacitidine | 0.249 | Antimetabolite | Antimetabolite | Unspecified |
| Trimetrexate | 0.203 | Antimetabolite | Antimetabolite | Antimetabolite |
| Lometrexol | 0.192 | Antimetabolite | Antimetabolite | Unspecified |
| Pentostatin | 0.187 | Antimetabolite | Antimetabolite | Unspecified |
| Zebularine | 0.176 | Antimetabolite | Antimetabolite | Unspecified |
| Doxofluridine | 0.17 | Antimetabolite | Antimetabolite | Unspecified |
| L-Alanosine | 0.139 | Antimetabolite | Antimetabolite | Unspecified |
| Tozasertib | 0.873 | Aurora kinase inhibitor | Aurora kinase inhibitor | Aurora kinase inhibitor |
| Alisertib | 0.788 | Aurora kinase inhibitor | Aurora kinase inhibitor | Aurora kinase inhibitor |
| Barasertib | 0.78 | Aurora kinase inhibitor | Aurora kinase inhibitor | Aurora kinase inhibitor |
| MK-5108 | 0.723 | Aurora kinase inhibitor | Aurora kinase inhibitor | Aurora kinase inhibitor |
| MLN-8054 | 0.619 | Aurora kinase inhibitor | Aurora kinase inhibitor | Aurora kinase inhibitor |
| TAK-901 | 0.551 | Aurora kinase inhibitor | Aurora kinase inhibitor | Aurora kinase inhibitor |
| JNJ-7706621 | 0.52 | Aurora kinase inhibitor | Aurora kinase inhibitor | Aurora kinase inhibitor |
| Dinaciclib | 0.95 | CDK inhibitor | CDK inhibitor | CDK inhibitor |
| Alvocidib | 0.692 | CDK inhibitor | CDK inhibitor | CDK inhibitor |
| BMS-387032 | 0.534 | CDK inhibitor | CDK inhibitor | CDK inhibitor |
| PHA-793887 | 0.457 | CDK inhibitor | Proteasome inhibitor | Proteasome inhibitor |
| AT-7519 | 0.375 | CDK inhibitor | CDK inhibitor | CDK inhibitor |
| Palbociclib | 0.21 | CDK inhibitor | HDAC inhibitor | HDAC inhibitor |
| Panobinostat | 0.92 | HDAC inhibitor | HDAC inhibitor | HDAC inhibitor |
| Quisinostat | 0.871 | HDAC inhibitor | HDAC inhibitor | HDAC inhibitor |
| Abexinostat | 0.621 | HDAC inhibitor | HDAC inhibitor | HDAC inhibitor |
| S-HDAC-42 | 0.609 | HDAC inhibitor | HDAC inhibitor | HDAC inhibitor |
| Belinostat | 0.547 | HDAC inhibitor | HDAC inhibitor | HDAC inhibitor |
| Entinostat | 0.545 | HDAC inhibitor | HDAC inhibitor | HDAC inhibitor |
| Chidamide | 0.527 | HDAC inhibitor | HDAC inhibitor | HDAC inhibitor |
| Vorinostat | 0.503 | HDAC inhibitor | HDAC inhibitor | HDAC inhibitor |
| OSU-HDAC-44 | 0.473 | HDAC inhibitor | HDAC inhibitor | HDAC inhibitor |
| Tacedinaline | 0.391 | HDAC inhibitor | HDAC inhibitor | HDAC inhibitor |
| CBHA | 0.37 | HDAC inhibitor | HDAC inhibitor | HDAC inhibitor |
| Pyroxamide | 0.324 | HDAC inhibitor | HDAC inhibitor | HDAC inhibitor |
| Tubastatin A | 0.242 | HDAC inhibitor | HDAC inhibitor | HDAC inhibitor |
| Pivanex | 0.183 | HDAC inhibitor | Antimetabolite | Unspecified |
| AT13387 | 0.856 | HSP90 inhibitor | HSP90 inhibitor | HSP90 inhibitor |
| Tanespimycin | 0.84 | HSP90 inhibitor | HSP90 inhibitor | HSP90 inhibitor |
| Ganetespib | 0.84 | HSP90 inhibitor | HSP90 inhibitor | HSP90 inhibitor |
| BIIB021 | 0.809 | HSP90 inhibitor | HSP90 inhibitor | HSP90 inhibitor |
| PU-H71 | 0.7 | HSP90 inhibitor | HSP90 inhibitor | HSP90 inhibitor |
| NVP-BEP800 | 0.466 | HSP90 inhibitor | HSP90 inhibitor | HSP90 inhibitor |
| KW-2478 | 0.378 | HSP90 inhibitor | HSP90 inhibitor | HSP90 inhibitor |
| CCT 018159 | 0.299 | HSP90 inhibitor | HSP90 inhibitor | HSP90 inhibitor |
| Geldanomycin | 0.295 | HSP90 inhibitor | HSP90 inhibitor | Unspecified |
| Colchicine | 0.977 | Microtubule inhibitor | Microtubule inhibitor | Microtubule inhibitor |
| Nocodazole | 0.964 | Microtubule inhibitor | Microtubule inhibitor | Microtubule inhibitor |
| Indibulin | 0.916 | Microtubule inhibitor | Microtubule inhibitor | Microtubule inhibitor |
| Vinblastine | 0.858 | Microtubule inhibitor | Microtubule inhibitor | Microtubule inhibitor |
| D-64131 | 0.712 | Microtubule inhibitor | Microtubule inhibitor | Microtubule inhibitor |
| Vincristine | 0.691 | Microtubule inhibitor | Microtubule inhibitor | Microtubule inhibitor |
| Vinorelbine | 0.669 | Microtubule inhibitor | Microtubule inhibitor | Microtubule inhibitor |
| Rotenone | 0.599 | Microtubule inhibitor | Microtubule inhibitor | Microtubule inhibitor |
| Vinflunine ditartrate | 0.589 | Microtubule inhibitor | Microtubule inhibitor | Microtubule inhibitor |
| Podophyllotoxin | 0.515 | Microtubule inhibitor | Microtubule inhibitor | Microtubule inhibitor |
| ABT-751 | 0.4 | Microtubule inhibitor | Microtubule inhibitor | Microtubule inhibitor |
| Cabazitaxel | 0.99 | Microtubule stabilizer | Microtubule stabilizer | Microtubule stabilizer |
| Epothilone B | 0.989 | Microtubule stabilizer | Microtubule stabilizer | Microtubule stabilizer |
| Docetaxel | 0.987 | Microtubule stabilizer | Microtubule stabilizer | Microtubule stabilizer |
| Paclitaxel | 0.975 | Microtubule stabilizer | Microtubule stabilizer | Microtubule stabilizer |
| Rigosertib | 0.98 | PLK1 inhibitor | Microtubule inhibitor | Microtubule inhibitor |
| MLN0905 | 0.976 | PLK1 inhibitor | PLK1 inhibitor | PLK1 inhibitor |
| GSK461364 | 0.963 | PLK1 inhibitor | PLK1 inhibitor | PLK1 inhibitor |
| Volasertib | 0.963 | PLK1 inhibitor | PLK1 inhibitor | PLK1 inhibitor |
| BI 2563 | 0.962 | PLK1 inhibitor | PLK1 inhibitor | PLK1 inhibitor |
| TAK-960 | 0.194 | PLK1 inhibitor | Antimetabolite | Unspecified |
| Carfilzomib | 0.968 | Proteasome inhibitor | Proteasome inhibitor | Proteasome inhibitor |
| Bortezomib | 0.966 | Proteasome inhibitor | Proteasome inhibitor | Proteasome inhibitor |
| Delanzomib | 0.961 | Proteasome inhibitor | Proteasome inhibitor | Proteasome inhibitor |
| Oprozomib | 0.93 | Proteasome inhibitor | Proteasome inhibitor | Proteasome inhibitor |
| Ixazomib | 0.822 | Proteasome inhibitor | Proteasome inhibitor | Proteasome inhibitor |
| MLN 2238 | 0.657 | Proteasome inhibitor | Proteasome inhibitor | Proteasome inhibitor |
| PSI | 0.624 | Proteasome inhibitor | Proteasome inhibitor | Proteasome inhibitor |
| MG 132 | 0.39 | Proteasome inhibitor | Proteasome inhibitor | Proteasome inhibitor |
| Dactinomycin | 0.683 | Protein synthesis inhibitor | Protein synthesis inhibitor | Protein synthesis inhibitor |
| Anisomycin | 0.554 | Protein synthesis inhibitor | Protein synthesis inhibitor | Protein synthesis inhibitor |
| Puromycin | 0.468 | Protein synthesis inhibitor | Protein synthesis inhibitor | Protein synthesis inhibitor |
| Mithramycin A | 0.46 | Protein synthesis inhibitor | CDK inhibitor | CDK inhibitor |
| Cycloheximide | 0.341 | Protein synthesis inhibitor | HDAC inhibitor | Unspecified |
| Idarubicin | 0.983 | Topoisomerase inhibitor | Topoisomerase inhibitor | Topoisomerase inhibitor |
| Daunorubicin | 0.979 | Topoisomerase inhibitor | Topoisomerase inhibitor | Topoisomerase inhibitor |
| Mitoxantrone | 0.974 | Topoisomerase inhibitor | Topoisomerase inhibitor | Topoisomerase inhibitor |
| Camptothecin | 0.96 | Topoisomerase inhibitor | Topoisomerase inhibitor | Topoisomerase inhibitor |
| Doxorubicin | 0.951 | Topoisomerase inhibitor | Topoisomerase inhibitor | Topoisomerase inhibitor |
| Teniposide | 0.922 | Topoisomerase inhibitor | Topoisomerase inhibitor | Topoisomerase inhibitor |
| Epirubicin | 0.912 | Topoisomerase inhibitor | Topoisomerase inhibitor | Topoisomerase inhibitor |
| Amsacrine hydrochloride | 0.833 | Topoisomerase inhibitor | Topoisomerase inhibitor | Topoisomerase inhibitor |
| Valrubicin | 0.8 | Topoisomerase inhibitor | Topoisomerase inhibitor | Topoisomerase inhibitor |
| Topotecan | 0.738 | Topoisomerase inhibitor | Topoisomerase inhibitor | Topoisomerase inhibitor |
| Etoposide | 0.577 | Topoisomerase inhibitor | Topoisomerase inhibitor | Topoisomerase inhibitor |
| Irinotecan | 0.39 | Topoisomerase inhibitor | Topoisomerase inhibitor | Topoisomerase inhibitor |
| Mytomycin | 0.89 | Unspecified | Topoisomerase inhibitor | Topoisomerase inhibitor |
| Thapsigargin | 0.85 | Unspecified | Proteasome inhibitor | Proteasome inhibitor |
| Ouabain | 0.85 | Unspecified | Microtubule inhibitor | Unspecified |
| FdCyd | 0.75 | Unspecified | Antimetabolite | Antimetabolite |
| Staurosporine | 0.72 | Unspecified | Aurora kinase inhibitor | Unspecified |
| Brefeldin A | 0.69 | Unspecified | HDAC inhibitor | Unspecified |
| Auranofin | 0.62 | Unspecified | CDK inhibitor | Unspecified |
| Obatoclax | 0.62 | Unspecified | HSP90 inhibitor | Unspecified |
| Bleomycin sulfate | 0.51 | Unspecified | Topoisomerase inhibitor | Topoisomerase inhibitor |
| Everolimus | 0.47 | Unspecified | Antimetabolite | Antimetabolite |
| BIX 01294 | 0.44 | Unspecified | HSP90 inhibitor | Unspecified |
| Melphalan | 0.42 | Unspecified | Aurora kinase inhibitor | Aurora kinase inhibitor |
| RAF-265 | 0.42 | Unspecified | Antimetabolite | Unspecified |
| Pictilisib | 0.38 | Unspecified | HDAC inhibitor | Unspecified |
| BIBR-1532 | 0.38 | Unspecified | Protein synthesis inhibitor | Protein synthesis inhibitor |
| Bardoxolone methyl | 0.38 | Unspecified | CDK inhibitor | Unspecified |
| Chlorambucil | 0.37 | Unspecified | HSP90 inhibitor | HSP90 inhibitor |
| 1-deazaadenosine | 0.37 | Unspecified | Protein synthesis inhibitor | Protein synthesis inhibitor |
| Ionomycin calcium salt | 0.37 | Unspecified | HDAC inhibitor | HDAC inhibitor |
| Thiotepa | 0.36 | Unspecified | Antimetabolite | Antimetabolite |
| Sunitinib | 0.35 | Unspecified | Aurora kinase inhibitor | Aurora kinase inhibitor |
| Temsirolimus | 0.32 | Unspecified | Antimetabolite | Unspecified |
| PLX-4720 | 0.31 | Unspecified | Antimetabolite | Unspecified |
| Golgicide | 0.3 | Unspecified | HDAC inhibitor | HDAC inhibitor |
| RO-5126766 | 0.3 | Unspecified | HSP90 inhibitor | Unspecified |
| IMAC2 | 0.3 | Unspecified | HSP90 inhibitor | Unspecified |
| Decitabine | 0.3 | Unspecified | Antimetabolite | Antimetabolite |
| Sirolimus | 0.29 | Unspecified | Antimetabolite | Unspecified |
| Hydroxychloroquine sulfate | 0.27 | Unspecified | Antimetabolite | Unspecified |
| Mitoguazone | 0.24 | Unspecified | HSP90 inhibitor | Unspecified |
| IMAC1 | 0.24 | Unspecified | HDAC inhibitor | Unspecified |
| Sorafenib | 0.22 | Unspecified | HSP90 inhibitor | Unspecified |
| Altretamine | 0.21 | Unspecified | Antimetabolite | Unspecified |
| Sodium phenylacetate | 0.21 | Unspecified | Antimetabolite | Unspecified |
| DFMO | 0.21 | Unspecified | Antimetabolite | Unspecified |
| Pifithrin mu | 0.2 | Unspecified | Antimetabolite | Unspecified |
| Erlotinib hydrochloride | 0.2 | Unspecified | Antimetabolite | Unspecified |
| Wortmannin | 0.2 | Unspecified | HDAC inhibitor | Unspecified |
| Lomustine | 0.19 | Unspecified | Antimetabolite | Unspecified |
| Ifosfamide | 0.19 | Unspecified | Antimetabolite | Unspecified |
| AT-406 | 0.19 | Unspecified | Antimetabolite | Unspecified |
| ABT737 | 0.19 | Unspecified | Antimetabolite | Unspecified |
| MDIVI-1 | 0.18 | Unspecified | Antimetabolite | Unspecified |
| Navitoclax | 0.18 | Unspecified | Antimetabolite | Unspecified |
| Busulfan | 0.17 | Unspecified | Antimetabolite | Unspecified |
| Streptozotocin | 0.16 | Unspecified | Antimetabolite | Unspecified |
| RU360 | 0.16 | Unspecified | Antimetabolite | Unspecified |
| DNP | 0.15 | Unspecified | Antimetabolite | Unspecified |
| Nimustine | 0.14 | Unspecified | Antimetabolite | Unspecified |

*PA = Phenotypic activity, ranging from 0 (induced phenotypes indistinguishable from untreated cells) to 1 (induced phenotypes completely distinguishable from untreated cells).
