# Supplementary material for: Robust Classification of Small-Molecule Mechanism of Action Using a Minimalist High-Content Microscopy Screen and Multidimensional Phenotypic Trajectory Analysis
Source: PLoS One. 2016 Feb 17;11(2):e0149439. doi: 10.1371/journal.pone.0149439 (PMC4757101; doi:10.1371/journal.pone.0149439)
Supplement: S4 Table — (DOCX) [file pone.0149439.s006.docx]

**S4 Table. List of all 8 test compounds, their published mechanism, the vendor that provided them, and the top concentration used in the assay.**

| Drug | Published Mechanism | Vendor | Top Concentration (μM) |
| --- | --- | --- | --- |
| Bortezomib | Proteasome inhibitor | LC Laboratories | 61 |
| Carfilzomib | Proteasome inhibitor | ChemieTek | 55 |
| Ixazomib | Proteasome inhibitor | Selleck Chemicals | 86 |
| MG-132 | Proteasome inhibitor | Torcris Bioscience | 47 |
| Gambogic Acid | NF-κB pathway inhibitor | MicroSource Discover Systems | 56 |
| Gossypol | Apoptotic agent | Selleck Chemicals | 56 |
| ICG-001 | Wnt/β-catenin pathway inhibitor | Selleck Chemicals | 61 |
| YM-155 | Survivin inhibitor | ChemieTek | 50 |
